# Conspiracy of Silence in Head and Neck Cancer Diagnosis: A Scoping Review

**DOI:** 10.3390/dj12070214

**Published:** 2024-07-11

**Authors:** Cristina Saldivia-Siracusa, Erison Santana Dos Santos, Wilfredo Alejandro González-Arriagada, Ana Carolina Prado-Ribeiro, Thaís Bianca Brandão, Adepitan Owosho, Marcio Ajudarte Lopes, Joel B. Epstein, Alan Roger Santos-Silva

**Affiliations:** 1 Departamento de Diagnóstico Oral, Faculdade de Odontologia de Piracicaba (FOP), Universidade Estadual de Campinas (UNICAMP), São Paulo 13414-903, Brazil; c234721@dac.unicamp.br (C.S.-S.); e228180@dac.unicamp.br (E.S.D.S.); carol_pr@yahoo.com.br (A.C.P.-R.); malopes@fop.unicamp.br (M.A.L.); 2 Faculty of Dentistry, University of Los Andes, Santiago 26183000, Chile; wgonzalez@uandes.cl; 3 Oral Medicine Service, Sírio Libanês Hospital, São Paulo 01308-050, Brazil; 4 Dental Oncology Service, São Paulo State Cancer Institute (ICESP-FMUSP), São Paulo 01246-903, Brazil; thais.brandao@icesp.org.br; 5 Departments of Diagnostic Sciences, Department of Otolaryngology—Head & Neck Surgery and Bioscience Research, College of Dentistry, The University of Tennessee Health Science Center, Memphis, TN 38163, USA; aowosho@uthsc.edu; 6 City of Hope Comprehensive Cancer Center, Duarte, CA 91010, USA; jepstein@coh.org; 7 Cedars-Sinai Medical Center, Los Angeles, CA 90048, USA

**Keywords:** disclosure, diagnosis, head and neck cancer, scoping review

## Abstract

Cancer disclosure represents a complex healthcare dynamic. Physicians or caregivers may be prompted to withhold diagnosis information from patients. This study aims to comprehensively map and synthesize available evidence about diagnosis nondisclosure regarding head and neck cancer (HNC) patients. Following the Joanna Briggs Institute guidelines, a scoping review was conducted across major databases without period restriction, yielding 9238 publications. After screening and selection, a descriptive synthesis was conducted. Sixteen studies were included, primarily conducted in academic settings (75%) from Europe and Asia, with a total population of 662 patients predominantly diagnosed with brain, oral, pharyngeal, or laryngeal tumors. Remarkably, 22.51% of patients were unaware of their diagnosis. Although physicians were the main source of diagnostic information (35%), they reported to often use vague terms to convey malignancy. Additionally, 13.29% of patients were aware of their diagnosis from sources other than doctors or caregivers. Caregivers (55%) supported diagnosis concealment, and physicians tended to respect family wishes. A high diagnosis-to-death interval, education, and age significantly influenced diagnosis disclosure. HNC patients expressed a desire for personalized open communication. Multiple factors influenced the decision on diagnosis disclosure. Current evidence on this topic varies significantly, and there is limited research on the consequences of nondisclosure. These findings reflect the underestimation of the patients’ outlook in the diagnosis process and highlight the need for further research, aiming to establish open communication and patient autonomy during the oncological journey.

## 1. Introduction

Disclosing a cancer diagnosis is a complex yet essential process in healthcare settings impacting professionals, patients, and caregivers/family members [1,2,3]. Awareness of one’s illness entails life-altering information, with diverse psycho-emotional repercussions in daily life and implications for therapeutic decisions and long-term survivorship considerations [4]. This phenomenon holds particular significance in head and neck cancer (HNC), where individuals face unique disease-related consequences. Long-term survivors may contend with significant physical disabilities, affecting essential functions such as breathing, eating, speaking, disfigurement, pain, and depression [2,5,6], especially in late diagnosis, which is a common situation that is particularly expected to keep rising after the COVID-19 pandemic [7,8].

In brief, “Conspiracy of silence”, also known as nondisclosure, or collusion, can be defined as the tacit or explicit agreement among family members and/or healthcare professionals to manipulate the information disclosed to the patient. This arrangement involves concealing aspects that shape the patient’s perception of their disease, sometimes without consent, such as cancer diagnosis, prognosis, or the gravity of the situation, to avoid conveying negative emotions or providing unrealistic optimism. This practice is often justified by the desire to reduce distress or anxiety associated with impending mortality, protect the family from emotional strain, and prevent disruptive emotional reactions [9,10]. The debate on whether patients should be informed about their cancer diagnosis has been extensively explored in cancer communication literature, mainly through questionnaire-based studies [5,11,12,13]. However, prior cross-sectional studies consulting professionals involved in the delivery of bad news have underscored that the physicians can dread patients’ negative emotions, yet proficient communication positively influences the mental health of professionals and patients [3].

Currently, there is limited literature on the impact of diagnosis nondisclosure on patients with cancer in the head and neck (HN) region. Herein, a scoping review (ScR) using relevant topic-related keywords and following the Preferred Reporting Items for Systematic Reviews and Meta-Analyses extension for Scoping Reviews (PRISMA-ScR) guidelines [14] was conducted across major scientific databases and gray literature to systematically map and synthesize the available evidence on this subject, encompassing study characteristics, cancer type, patient preferences, patient issues, and consequences associated with the conspiracy of silence involving these patients. This review also aimed to identify gaps in current scientific knowledge related to this matter, striving to obtain findings that positively impact current healthcare practice and policy concerning patient disclosure.

## 2. Materials and Methods

### 2.1. Study Design, Protocol, and Registration

A preliminary search of the literature was conducted across major databases (PubMed, Web of Science, and PROSPERO) using the keywords “conspiracy of silence”, “diagnostic disclosure”, and “head and neck cancer”, and no current or underway systematic or scoping reviews on the topic were identified. Due to the theme’s nature, we opted for a scoping review rather than a systematic review to enhance literature mapping and offer a synthesized background overview of unexplored evidence in the context of cancer in the HN region. The methodology adhered to the Joanna Briggs Institute (JBI) Reviewer’s manual [15], with each step following the Preferred Reporting Items for Systematic Reviews and Meta-Analyses extension for Scoping Reviews (PRISMA-ScR) guidelines [14] (Online Resource S1). Our research design protocol was registered on the Open Science Framework (OSF; https://osf.io/aq58c, accessed on 6 February 2024). Our ScR followed the Population, Concept, and Context (PPC) outline, identifying key points as follows: participants/population—cancer patients, main concept—diagnostic disclosure, as defined by the included studies, and context—HN oncology. This review aimed to address issues specific to the conspiracy of silence in HNC, guided by the following research questions:

- What is known about this concept?
- What are the preferences, issues, and motives encountered by individuals, caregivers, and healthcare providers related to the conspiracy of silence in HNC patients?
- What are the consequences that HNC patients experience in relation to the management of their diagnostic disclosure?

### 2.2. Eligibility Criteria

Peer-reviewed journal papers were included if they met all the following criteria: (a) studies that assessed diagnostic disclosure involving patients with solid tumors of the HN region (including brain, eye, and thyroid), as well as licensed, specialized HN health providers (doctors, dentists, and specialists), and/or the patients’ caregivers (family/friends (paid or unpaid), as defined by the included articles), (b) patients > 18 years of age, (c) English, Spanish, or Portuguese language, and (d) had available sociodemographic information.

Papers were excluded if they (a) did not fit into the conceptual framework of the study or (b) focused on ethically vulnerable groups, such as pediatric patients or pediatric relatives, as well as on nurses and/or students/residents. Further, if they (c) assessed hematologic malignant diseases if they were not described as solid tumors, (d) included patients with neurodevelopmental, cognitive, disruptive, and dissocial disorders, considered as disabilities, or (e) focused on any other disclosure information that did not involve solid HN tumor diagnosis, such as conspiracy of silence regarding genomic risk/susceptibility, genetic test results, screening programs, and/or imaging results. They were additionally excluded if they were (f) studies regarding diagnostic information delivered through telehealth consultation, (g) studies regarding diagnostic nondisclosure of metastasis, second primary tumors, recurrences, treatment failure, treatment changes, palliative care transition, or prognosis (exclusively), (h) systematic and narrative reviews, protocols, conference abstracts, opinions, editorials, short communications, letters, news, duplicate populations (keeping the most recent), in vitro or animal studies, case reports, and case series, (i) papers with HNC patients’ data which were not possible to extract from the total population, and (j) unavailable full text.

### 2.3. Types of Sources

Observational studies were eligible for inclusion. No restrictions regarding the time of publication were applied.

### 2.4. Information Sources and Search Strategy

The article search and selection were executed collaboratively by two authors (C.S.-S. and E.S.D.S.). On 14 December 2023, the following bibliographic databases were queried: PubMed, Embase, SCOPUS, Web of Science, Latin American and Caribbean Health Sciences (LILACS), and the Cochrane Library. Additionally, a manual exploration was performed on Google Scholar (first 200 articles obtained through the applied search strategy), ProQuest, and reference lists of the included articles (possible topic-related papers) to identify any eligible documents not retrieved electronically. The search strategy details are available in Online Resource S2.

### 2.5. Selection of Sources of Evidence

Following the initial search, the two reviewers independently carried out and cross-verified the selection process in a consensus meeting. After the search was performed, all citations were input into EndNote X7 (Clarivate Analytics, Philadelphia, PA, USA), and duplicate records were eliminated. Rayyan QCRI served as a reference manager for manual exclusion of duplicates, identification of relevant articles through title and abstract review, screening, eligibility assessment based on predefined criteria, and documentation of primary reasons for exclusion. Discrepancies were resolved through discussion and, if necessary, consultation with a third author (A.R.S.-S.). Subsequently, data extraction was performed by the primary researcher and reviewed by a second author.

### 2.6. Bibliometric Analysis

Bibliometric analyses regarding the impact factor in Journal Citation Reports (JCR) for 2022 and Google Scholar, Web of Science, and Scopus citations were performed on 25 January 2024.

### 2.7. Data Collection and Charting

To extract the pertinent information from the included sources of evidence, two reviewers (C.S.-S. and E.S.D.S.) collaboratively devised a comprehensive data charting form, extracting as much available information as possible to determine the relevant variables. The selected articles were subsequently analyzed for key data points, including author(s), year of publication, country, objective, study design, eligibility criteria, study population, total number of disclosed cases, location, disclosure of diagnosis, reported perspectives and contextual factors, number of disclosed and undisclosed participants, prevalence of collusion, and outcomes of any formal assessment of engagement (e.g., attitudes, beliefs, knowledge, benefits, unintended consequences), along with key findings and details on how outcomes were measured. Data collection items were as follows: (1) study characteristics (2) population characteristics, (3) disclosure assessment, and (4) main results. Both qualitative and quantitative data were tabulated and processed in Microsoft Excel^®^, version 2110.

### 2.8. Risk-of-Bias Assessment

The same two reviewers who conducted the study selection and data collection (C.S.-S. and E.S.D.S.) assessed the risk of bias of the included studies using the Joanna Briggs Institute Critical Appraisal Checklist tools according to the type of study: analytical cross-sectional studies, cohort studies, and case-control studies. For this purpose, both authors answered each question of the checklists independently, choosing between “yes” (green), “no” (red), “unclear” (yellow), and “not applicable” (gray) options. Discrepancies were resolved through discussion and, if necessary, consultation with a third author (ARSS). Finally, studies were categorized as follows: “High risk of bias” when the study reached a score between 0–49% “yes” answers, “Moderate risk of bias” when the study reached a score between 50–69% “yes” answers, and “Low risk of bias” when the study reached a score between 70–100% “yes” answers.

### 2.9. Synthesis of Results and Statistical Analysis

The results were analyzed based on information extracted from the included studies. The consistency of the data relied entirely on the available information. Common extracted data were categorized for comparison and analysis. A narrative, descriptive synthesis covering the findings of the studies is presented, using values of frequency, mean, and average, as possible. Graphs and tables were constructed using Microsoft Excel (Microsoft Corporation, Redmond, WA, USA) to illustrate the results. Considering the heterogeneity of the compiled data, quantitative analysis was not performed. During the preparation of this work, DeepL Write and ChatGPT tools were used by the authors in order to improve readability. The authors reviewed and edited the content as needed and take full responsibility for the content of the publication.

## 3. Results

### 3.1. Selection of Evidence Sources

**Figure 1:**
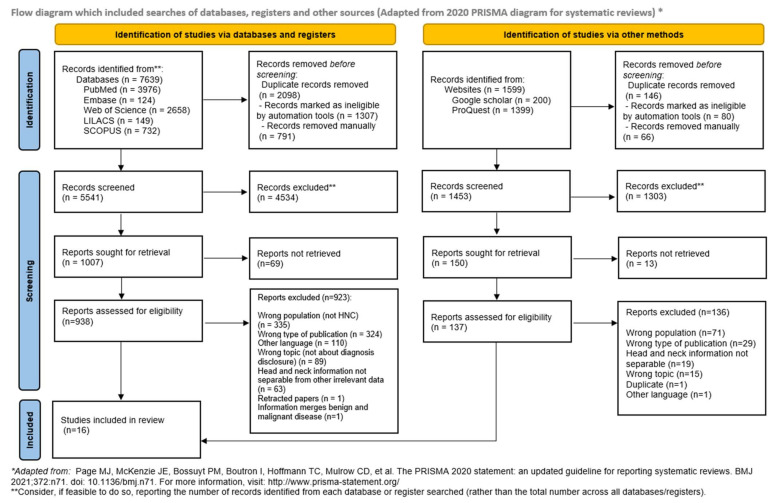
Flow diagram of the literature search and selection criteria [16].

The initial search yielded a total of 9238 records. Of those, 7639 records were identified in the main databases; after duplicate removal, 5541 records remained. After title and abstract review, 4534 records were excluded, leaving 938 records for eligibility assessment. Full-text reading led to the exclusion of 923 reports. Regarding gray literature, 1599 records were identified, 1453 remained after duplicate removal, and 137 were assessed for full-text eligibility, resulting in the exclusion of 136 records. Ultimately, 16 studies were included in this ScR (Figure 1).

### 3.2. Bibliometric Analysis

All studies were published within the last 30 years (range: 1997–2020), with 10 (66.66%) published within in the past 15 years. Geographically, Europe and Asia dominated the worldwide distribution analysis, with six (37.5%) studies each, followed by America with two (12.5%), and Africa (6.25%) and Oceania (6.25%) with one study each (Table 1).

Two journals had multiple publications: *Supportive Care in Cancer* with three and *Psycho-Oncology* with two. The mean impact factor of publishing journals was 5.94 (range: 0–50.5). The mean number of citations per study was 44.37 in Google Scholar (range: 6–122), 22.87 in Web of Science (range: 0–73), and 20.37 in Scopus (range: 0–75; Figure 2).

### 3.3. Characteristics of the Sources of Evidence

Table 1 presents the main sample characteristics collected from the 16 included studies. Fourteen (87.5%) publications were retrospective and two (12.5%) were prospective.

Twelve (75%) studies were performed at universities [2,5,6,10,18,19,21,22,23,24,25,26] and four (25%) were performed outside academic institutions [11,12,17,20]. The use of questionnaires constituted the most prevalent method of assessment, employed exclusively in nine (56.25%) studies [2,5,12,17,18,21,22,23,24,26]. Nine (56.25%) of the included studies exclusively considered HN patients as their population [2,5,6,12,18,19,21,22,23]. In contrast, three studies (18.75%) focused on patients along with their caregivers [20,25,27]. Two (12.5%) studies included only physicians [11,26], and one (6.25%) assessed a group comprising both physicians and caregivers [17]. Additionally, one study (6.25%) evaluated patients, their physicians, and their caregivers [24].

According to the 14 (87.5%) studies assessing patients, information confirmed a total of 662 individuals. Sample sizes varied from 16 to 151, with a mean of 41.37 patients per study (Table 1). From these, studies from China (n = 151) [10] and France (n = 91) [21] represented the biggest sample, and USA (n = 16) [2] and Nigeria (n = 17) [23] constituted the countries of the studies with the smallest samples. Of these participants, 300 were male and 175 were female, while 5 (31.25%) studies did not report sex information [12,17,19,23,24]. The patients’ mean age was 56.65 years, as reported in 8 (50%) studies (range: 20–85 years) [2,5,6,10,18,21,22,25]. Regarding tumor localization, most studies (62.50%) assessed patients with tumors located in the upper aerodigestive tract (including the oral and maxillofacial region, pharynx, and larynx) or brain (Online Resource S3).

The primary objectives, key points, and outcomes analyzed in each study are summarized in Table 2. Most of the studies addressed more than one objective. The most common objective of the included studies was to evaluate the perspectives, preferences, and/or requests of the patients regarding diagnosis disclosure [2,5,12,18,19,20,22,23,24], comprising a total of nine (56.25%) studies.

### 3.4. Critical Appraisal within Sources of Evidence

After risk-of-bias assessment, we classified a total of five studies as low risk (three cross-sectional, one cohort, and one case-control studies), four studies as moderate risk, and seven as high risk of bias (Online Resources S4 and S5). The main influencing factor for bias risk was the inability to use objective, standardized methods to measure the assessed outcomes, as questionnaires and interviews intrinsically represent subjective evaluations.

### 3.5. Results of Sources of Evidence and Data Synthesis

#### 3.5.1. Definition

Delivery of information was mostly declared as diagnostic disclosure. While some studies did not use a specific term, they referred to “revealing the presence of malignancy” [11], “clearly informing the patient” [17], or “bad news communication” [18]. Only two (12.5%) studies provided a clear definition of disclosure [10,17]: Costantini et al. defined diagnostic disclosure as using words such as “cancer”, “malignant tumor”, or “neoplasm” when delivering diagnostic information. Additionally, patients were considered informed if they knew about their diagnosis, regardless of who provided the information [17]. Wang et al. defined patients’ awareness of cancer diagnosis as knowing that their illness was cancer [10]. No further information was reported on this matter.

#### 3.5.2. Perspective of the Patient

Overall, 513 patients (77.49% of the total sample) were aware of their diagnosis. In seven studies (43.75%), all patients were informed of their diagnosis, while in six studies (37.5%), both informed and uninformed patients were assessed. One study (6.25%) did not report whether patients were aware of their diagnosis (Table 1). Three (18.75%) studies reported the percentage of patients willing to receive information about their disease [5,12,24], ranging between 89% and 93.6% [12,24], and one (6.25%) study reported that 84.1% of patients were willing to receive more information than what was provided [5].

In 11 (68.75%) studies, information regarding who disclosed the diagnosis was obtained from 348 patients [2,5,10,12,17,18,19,21,22,23,25]. According to the available information, a total of 232 patients received diagnostic disclosure by a physician (35% of the total sample). Nevertheless, three (18.75%) studies claimed that physicians were the main providers of diagnostic information, but no quantitative values were reported [18,19,22]. From this group, one (6.25%) study reported that 80% of the whole sample was informed by physicians but was not specific for HNC [17], while another study (6.25%) indicated that most “bad news” was delivered by physicians and that 48% of patients were communicated bad news by physicians that they did not meet previously [18]. Another study (6.25%) claimed that diagnosis information was most often received directly from a physician during face-to-face appointments, but not always [22]. In addition, 4 (25%) studies demonstrated that, overall, caregivers gave the diagnosis to 28 patients (4.22% of the total sample) [2,10,12,17], with 1 (6.25%) study not reporting quantification [17]. Finally, 5 (31.25%) studies conveyed that a total of 88 (13.29% of the total sample) patients discovered their diagnosis through other means [10,12,17,21,22], such as nurses, fellow patients, overhearing conversations, changes in relatives’ behavior, reading medical records, or attending treatment. Regardless, two (12.5%) of those studies did not provide specific information about this claim [17,22]. Remarkably, 61.9% of patients obtained their own information in Wang’s research [10]. Also, one study reported that female patients more often obtained their information from their treating physician, while male patients more often received their information from another source [12].

While almost all included studies considered cognitive impairment as an exclusion criterion, a single study recalled impaired cognitive capacity as a hindering factor for disclosure understanding and a threat to conversations about the diagnosis in brain tumor patients [25].

#### 3.5.3. Preferences or Requirements

Eight (50%) studies investigated patient preferences, revealing nuanced aspects of their perceptions. Preferences about the provider of diagnostic information were reported in only one (6.25%) study [24], with 88.7% favoring doctors and 8.9% preferring a family member. In a single study, 95% of patients expressed a desire for doctors to inquire about their preferences about the amount of information to be delivered [18]. Concerning the disclosure process, two (12.5%) studies reported high patient satisfaction with information quality [2], time spent with physicians [21], and timing of disclosure [2,21], contrasting with another study (6.25%) reporting this result for only half of its patients [23]. Inconsistently, a study revealed that only two patients had positive experiences during diagnosis delivery [20]. Bad consultations were experienced when the physician seemed stressed, unprepared, dishonest, or showed unclear communication that failed to align with their needs and preferences according to the patient’s perception [22]. A unanimous patient preference for face-to-face disclosure with honesty about disease severity regardless of its nature was revealed [5,18]. Distressed patients and those with more severe malignant tumors reported heightened communication needs and demand for information [18]. Statistically significant results were obtained, showing that patients with brain tumors more often preferred shared decision-making with their physician [12].

Only one study (6.25%) acknowledged the outlook of patients seeking a second opinion after receiving diagnostic news, with a reported incidence of 9% of patients opting for this course of action [5].

#### 3.5.4. Perspective of the Physician

Specialists of various health areas were identified as being involved in the delivery of HN diagnosis news, such as otorhinolaryngology surgeons [11], oncologists [11], radiotherapists [11], neurosurgeons [11,18,21,26], dental surgeons [19], psychiatrists [6], and others [11,21,26]. Some studies only described these professionals as physicians [2,10,12,22], doctors [5,23,24], or health professionals [17].

Three (18.74%) studies provided insights into the physician’s perspective [11,24,26]. One study reported that 37% of surgeons would “most often than not” inform patients about the diagnosis, often using disguised information [11]. Conversely, other studies have indicated that 41.4% to 61.5% of surgeons are mandatory tellers [24,26]. Concerning caring for the relatives’ wishes, those findings also revealed that 45.7% of doctors would not disclose if the family objected, 67.6% of surgeons sought to avoid breaking bad news, and 41.7% believed informing the family before the patient was best [24], while another study claimed that more than half of the physicians respected their wishes when the family opposed disclosure [26]. According to previous studies with statistical analyses, one (6.25%) revealed the treating physician as the primary source of diagnostic information [12], while another (6.25%) showed that surgeons found it easier to provide information and handle patient emotions if they regularly disclosed diagnoses [11].

#### 3.5.5. Perspective of the Caregiver

Six (37.5%) studies specified who they considered relatives or caregivers, in which friends and family [2], spouses [10,25], and first-degree relatives [10,24] were considered as such. Two (12.5%) studies provided detailed definitions for this group, considering the closest and the most informed person about the last three months of the patient’s life, which resulted to be mostly female relatives (spouse or child) [17], or the person named by the patient as the most involved in their care [20].

Four (25%) studies explored caregivers’ preferences [6,20,24,25]. Among them, two (12.5%) studies reported the percentages of relatives (54.4% and 58%) supporting diagnosis concealment [6,24]. In one of these assessments, 54.6% of caregivers believed that informing relatives before the patient was best, however, only 6.4% of patients agreed [24]. Only one (6.25%) study reported caregivers’ feelings after disclosure, finding that all interviewed caregivers expressed shock and disbelief upon learning about the diagnosis [20]. A single (6.25%) study delved into family communication dynamics between spouses dealing with a cancer diagnosis, where spouses were typically less satisfied than patients with the provided information [25] (Table 2). No significant relationship was found through statistical analysis between the type of caregiver and the likelihood of a patient being informed about their diagnosis [17].

#### 3.5.6. Used Terms

The term “cancer” was used to determine the diagnosis in three studies (18.75%) [12,23,24]. However, words such as “disease” [5,12], “growth” [11,23], “mass” [12], “tumor” [23], and “injury” [12] were also used to talk about the disease. One study also reported that some physicians opted to use other unspecified terms or multiple terms [12]. Another study showed that 71.7% of the included patients wanted to hear the word “cancer” when receiving their diagnosis, but only 52.85% of doctors adhered to this statement [2]. Interestingly, one (6.25%) study assessed understanding of the patients’ diagnosis, showing that 31% made no mention of the malignant nature of their disease when asked [19].

#### 3.5.7. Influential Factors for Diagnosis Concealment

Concerning possible influential factors, six (37.5%) studies reported relevant information [5,10,11,12,17,26]. The following were considered as such in the HNC disclosure process: education level [2,17,18,19,20,23], age [11,12,17], gender [12,17,26], patient’s desire to know their diagnosis [11,26], type of cancer [12,17], mental intolerance [5], medical paternalism [5], intelligence [11], number of dependents [11], anxiety [11], cancer histological grade [26], family wishes [26], patient’s condition [26], religion [26], need for treatment [26], neuro-oncology policies [26], and geographical location [17]. Statistically significant associations were found between diagnostic disclosure and HN cancer [12,17], a greater diagnosis-to-death interval [17], education level [10,17], physicians working at facilities performing over 50 glioma cases per year, physicians in metropolitan areas, and those with additional psychosocial support systems for patients [26]. Conversely, the probability of being informed about the diagnosis significantly decreased with increasing age at death [17]. In other studies, no correlations were found between diagnosis awareness and patients’ age [5,10], sex [5,10], cancer type [10], disease stage [10], and hospital or residential location [10].

#### 3.5.8. Effects

Three (18.75%) studies delved into the psychological [18,19] and psychiatric [6] effects of diagnosis disclosure of cancer in the HN region through different assessment tools (Table 1), reporting rates ranging from 7% to 83.3% for depressive symptoms and 14% to 60% for anxiety levels [18,19]. Depression symptoms were more frequently observed among cancer patients [6,19]. Moreover, a significant increase in these symptoms was noted after cancer disclosure [19]. The overall prevalence rate of psychiatric disorders among patients with malignant diseases and scores of clinically relevant cancer-related distress were reported in one (6.25%) study each, yielding rates of 46% and 54%, respectively [6,18]. However, benign versus malignant diseases and informed versus uninformed groups within malignant diseases yielded nonsignificant results [6]. A single study (6.25%) assessed cognition status and revealed a 40% prevalence of cognitive impairment scores [22], another study (6.25%) reported no psychological consequences reported after disclosure, but they did not specify assessment methods [5], and only one (6.25%) study showed that role functioning was the most affected area in patients’ quality of life [22].

Only two (12.5%) studies reported the integration of patients with mental assistance and medical support systems [21,26], indicating that 27.4% to 28.6% of patients were monitored by a psychologist or social worker and connected with the oncology network, respectively. Moreover, 51.4% of patients cited the availability of patient support systems, including psychiatrists, psychosomatic physicians, or others [26].

## 4. Discussion

The delivery of bad medical news is an intricate process that represents a challenging task for the healthcare teams, patients, families, and caregivers [3]. This challenge is compounded by the fact that cancer remains a taboo subject in many places worldwide, a notion supported by diverse cultural and medical ethics principles [28,29]. Previous systematic reviews have assessed the topic of diagnostic collusion in oncology [9,28]. However, following a comprehensive literature review, we believe that this is the first scoping review on the conspiracy of silence in HNC patients.

Our evidence overview revealed an increased focus on diagnosis disclosure, since we found that studies on this topic were published in the last 30 years, reflecting a growing interest, likely linked to growing societal changes in recent times. The absence of publications may be particularly true in dental care, despite the relevance for dentists who may need to convey an oral cancer diagnosis [3]. This gap could result in oral health professionals missing valuable insights to improve clinical practice, considering their potential involvement in communicating distressing news to patients. Growing efforts to generate evidence and impart communication skills, accessible to both trainees and professionals, are important for professionals to confront these situations, as evidenced in recent studies [3,4,30].

We also recognized a relevant heterogeneity regarding the available literature: a great diversity in objectives, populations, and assessed outcomes was noted. Sample sizes varied greatly, which can limit the generalizability of some results. Additionally, while the implementation of questionnaires was consistent as a method of assessment used in most studies, these were also very diverse in nature, comprising a total of 13 different questionnaires applied, resulting in assorted obtained information. We must also recognize the limitation of using subjective evaluation tools, since they are subject to interpretation and perspective. In this sense, open questions, interview responses, and subjective appreciations are difficult to measure quantitively, hampering data extraction and statistical analysis, and introducing bias risk, which was confirmed through critical appraisal. The evaluation of confounding factors in these studies is also a challenge, as these studies assess psychological, behavioral, and emotional aspects of patients affected by various diseases that represent specific burdens to the patient’s quality of life depending on factors such as location, stage, and time of the diagnosis.

Some studies of the ScR showed that the proportion of patients aware of their diagnosis was higher compared to other areas of the body [12,17]. The HN area represents a region with significant peculiarities concerning how cancer impacts the patient. Its involvement can lead to intense physical consequences, cognitive and emotional dysfunction in patients with brain tumors, functional and cosmetic alterations, as well as notable psychological repercussions and emotional tolls [5,11,31]. The reported higher awareness is likely associated with subsequent effects of disease progression and treatment. Interestingly, scarce information has been reported regarding the impact of the side effects of this specific diagnosis [11].

The decision to withhold HNC diagnosis information may stem from the patient, but diverse beliefs about the potential consequences for the patient’s mental well-being may prompt doctors and caregivers to adopt the conspiracy of silence [9,32]. This tendency is notably observed in the Eastern World due to a longstanding paternalistic approach in medicine [6,24,28]. For instance, a previous study from Iran demonstrated that cancer patients would often be systematically referred to oncology services without knowing their diagnosis [33]. Another study reported that 80% of Chinese physicians might comply with families’ nondisclosure request, as compared with 10% of American doctors [34]. While a previous meta-analysis found higher numbers of studies in India, the USA, and China [9], our global review indicates that clinical and psychological impacts are widespread, with Europe being a prominent contributor. Even in the West, known for a more patient-centered care [35], resistance to delivering bad news is acknowledged, particularly in Southern European countries, but also in America [36,37]. A Brazilian study on oncology surgeons’ perspectives found a preference for using what they reported as a “kind lie” by omitting details [38]. Intriguingly, when considering themselves as patients, these surgeons expressed a preference for receiving comprehensive information [38], aligning with findings from some of the included studies [11].

Nonetheless, former studies outside the HN region have noted a decline in the preference for information concealment [17,25]. Still, we confirmed that a relevant 22.51% did not receive their diagnosis, agreeing with a 24.15% prevalence previously stated by a systematic review [9]. Since the included studies mostly comprised disclosed patients, there could be a bias causing an underestimation of the overall prevalence. However, the results of our ScR align with claims from various cancer-related studies, emphasizing the moral and ethical dilemmas faced by physicians when deciding whether to disclose, especially when the family expresses a preference for nondisclosure [39]. We identified diverse patient preferences, with a general desire for open communication. Notably, 30% of patients in one study experienced a mismatch between preferences and physicians’ behavior during diagnosis, associated with decreased satisfaction in received information but not in other psychosocial well-being aspects [18]. It has been suggested that prognostic discussions should be tailored to each patient’s needs [40]. In terms of diagnosis, communication should also be customized to strike a balance between patients’ specific needs and preferences rather than assuming nondisclosure, aiming to provide adequate information without overwhelming patients [9].

The principle of family beneficence poses an important barrier to truthful communication [39,41], with families often excluding patients and acting as communication intermediaries [42,43]. Supporters argue that concealing diagnostic information protects the patient, fosters hope, and prioritizes household well-being. A previous study already reported that 85.4% of the doctors said that the family usually asks the physician not to disclose the diagnosis to the patient [44]. However, efforts to hide the truth may not be effective [6], as some patients discover their diagnosis independently. Our analysis revealed a significant percentage of 13.29% patients who knew about their diagnosis through their own means. Lack of information can increase uncertainty, anxiety, and loneliness [18], potentially causing mistrust and affecting relationships [6,12]. This contrasts with studies outside the HN area, where appropriate information correlated positively with mental and global quality of life, and negatively with depression and anxiety [13]. There is limited evidence on relationships between diagnosis disclosure and quality of life [41]. Only one study that properly assessed quality of life in our targeted population was found, yielding minimal results. Further investigation into these effects could significantly enhance our current understanding.

The results of this ScR show that multiple terms were used to disclose HNC, such as “growth” [11] or “injury” [12]. This strategic ambiguity could be a form of information concealment [45]. Using less intimidating language to soften the delivery of bad news can cause patient misinterpretation, which can impact a patient’s perception and lead to a lack of awareness about their disease and its effects [40], as confirmed by an included study [19]. Nonetheless, communication strategies were not really explored. In this context, the only study that assessed communication methods showed a mandated-by-law disclosure process [21], a fact that may reflect the need for public policies that ensure patients’ rights to informed consent and clear information. Previous literature has reported multiple communication strategies to deliver bad news in healthcare environments, such as the ABCDE and the SPIKES protocols [46]. A previous systematic review demonstrated improved professional performance when using this latter tool [47], confirming that training within these strategies has a positive impact on physician practices [3]. Reports about the use of these strategies as well as reactions to these tools can further increase their use and help them adapt these tools to clinical care.

Several influential factors in diagnosis nondisclosure were assessed, with limited statistical associations found, particularly in relation to age, high education level, and the diagnosis-to-death interval. Notably, divergent results were obtained: while some reported no association with patient age [5,10], others reported that older patients were typically less informed [17]. This comes as no surprise, as current literature presents both similar and conflicting results [40,42,48,49]. Malmstrong et al. reported that participants generally wanted to know “the truth” about diagnosis and prognosis, but what they meant by “the truth” and how it should be communicated was highly variable [22]. Such heterogeneity observed across studies within and beyond the HN region suggests that multiple factors could influence the diagnosis disclosure decision, and they may significantly vary depending on the cultural, social, and personal contexts of the patient, caregivers, and healthcare professionals [22].

The conviction that full disclosure could negatively impact the patient’s psychological state is not uncommon, occasionally fearing depression and suicidality [6,26]. Former studies indicated that otolaryngology patients often experience higher levels of stress and depression compared to those with other medical conditions [50,51]. We found that, while the prevalence was variable, the included studies approaching this matter identified variable prevalence of depression and anxiety across the population. Notably, psychological care within the medical team during oncological treatment was considered in only two studies [21,26]. In this sense, supporting the patient’s mental well-being can be crucial as they navigate through these challenging circumstances.

Patient autonomy holds significant importance, aligning with robust ethical principles that underscore patients’ rights to information and involvement [38,41]. Diagnostic disclosure significantly influences decisions throughout the course of the patient’s treatment. In an included study, 9% of patients sought a second opinion, despite continuing with their initial medical care. Another included study reported that medical disclosure with the neurosurgeon occurred 11 days after surgery [21]. This highlights the limitations imposed on patients who are not informed. Hiding diagnostic information restricts their ability to consider factors such as secondary effects and prognosis, thus limiting active participation in their own medical care. While nondisclosure practices persist, evidence suggests that most patients prefer to be informed of their diagnosis and express a desire for more information, irrespective of cancer stage [41,42], which was also reflected in our obtained results. Honesty fosters patient collaboration and sets the stage for realistic expectations. Conversations about diagnosis should align with individual patient and family values, thereby enhancing societal support for patients with cancer in the HN region [12,32]. Research focusing on the subjective preferences of individuals living with the disease should be prioritized.

Patient, family, and caregiver support may be greatly facilitated by patients with “living wills”, statements of preferences in healthcare decision-making, and appointing healthcare representatives. The relevance of internet access and support groups with attending benefits and risks will change the discussion and guidance for disclosure of diagnosis, treatment, and prognosis. A component of disclosure may include recommendations for where to obtain more relevant data and support groups, local, national, and international, and avoiding potential misinformation that is common on the internet. This new “information” era may modify disclosure and details of diagnosis, treatment, and prognosis and may lead to improved patient care, but must be managed carefully and assessed in new studies.

Some limitations should be noted. Since all participants in these studies volunteered, the presented findings might not be comprehensively representative of the entire targeted population and could potentially reflect high-standard practices, even more so in academic settings. Moreover, relevant studies from non-English-speaking countries, particularly those embodying a specific paternalistic approach to cancer in Eastern cultures, might have been overlooked. The relevance of regional, societal, and broad psychosocial factors and the increasingly multicultural world impact expectations for disclosure and providing information, which is not clearly addressed in past studies. The selected studies primarily used questionnaires and interviews as assessment methods. While these qualitative subjective methods are commonly employed in studies focusing on social processes [25], they also carry a bias risk, which could be further heightened by the utilization of retrospective reports that can increase the risk of unconscious bias influenced by the participants’ perceptions. It is also crucial to highlight that several studies were excluded from this ScR due to inadequacies in the information related to the patients, which was either unreported, unclearly described, or unretrievable in the articles. Furthermore, even in the included studies, significant details regarding population characteristics and methodology were frequently lacking. Consequently, while some interesting findings from the included studies are presented in this review, they must be interpreted with caution.

## 5. Conclusions

Diagnostic disclosure remains understudied in head and neck oncology. The sixteen studies analyzed in this scoping review shed light on diagnostic disclosure practices, in which most patients received their diagnosis from physicians, and multiple factors influencing the decision to deliver a diagnosis were identified, affecting the healthcare team, the family, and the patient. The current ScR revealed a 22.51% prevalence of diagnostic collusion in patients with cancer in the HN region and comprehensively described the main issues, needs, and beliefs. Patients generally expressed a desire for accurate and detailed information about their diagnosis, tailored to their wants and needs. Additional studies on the consequences of diagnostic nondisclosure in the HN oncology population with diverse cultural backgrounds, studies on communication strategies and applicability for healthcare professionals’ training, and longitudinal studies tracking long-term outcomes after disclosure are encouraged to establish optimal clinical protocols, promote open communication, discover valuable insights, and develop evidence-based guidelines of information delivery in cancer care.

## Figures and Tables

**Figure 2:**
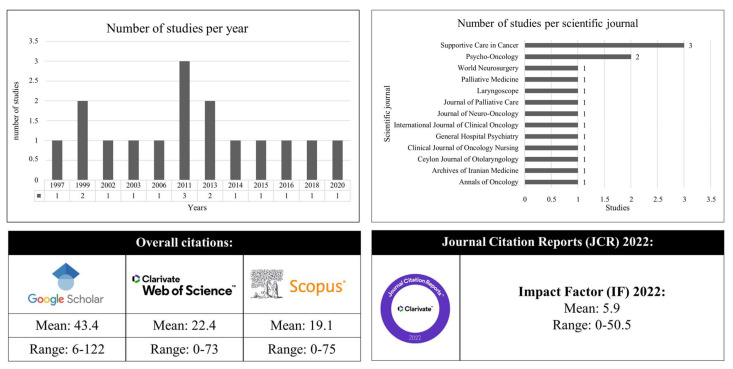
Bibliometric analysis.

**Table 1:** Main sample characteristics of the included studies.

Author (Year)	Location Setting	Study Design and Used Methods/Techniques	Study Population and Sample Size	Patient’s Awareness of the Disease
Burton, M.V. and Parker, R.W. (1997) [11]	National Health Service hospitals in two West Midlands cities, United Kingdom	Retrospective: evaluation through interviews	Physicians: 5 ear, nose, and throat surgeons and 3 neurosurgeons	NA
Costantini, M. et al. (2006) [17]	Unit of Clinical Epidemiology, National Cancer Institute, Genova, Italy	Retrospective: evaluation through application of a modified section of the Views of Informal Careers—Evaluation of Services (VOICES) questionnaire, from the Italian Survey of Dying of Cancer (ISDOC)	Physicians or caregivers from 28 patients *	21 (74%) patients received diagnosis disclosure
Goebel, S. and Mehdorn, H.M. (2018) [18]	Department of Clinical Psychology and Psychotherapy, Institute of Psychology, Christian Albrechts University, Kiel, Germany	Retrospective: evaluation through application of an original questionnaire (Measure of Patients’ Preferences)	42 patients	All patients received diagnosis disclosure
Graner, K.M. et al. (2015) [19]	Piracicaba Dental School, Campinas State University, São Paulo, Brazil	Retrospective: evaluation through application of a semi-structure interview and questionnaire (State-Trait Anxiety Inventory (STAI), the Beck Depression Inventory (BDI), and the Alcohol Use Disorders Identification Test (AUDIT))	29 patients	All patients received diagnosis disclosure
Hosaka, T. et al. (1999) [6]	Otolaryngological Surgery Department, Tokai University Hospital, Japan	Retrospective: evaluation through a DSM-III-R Structured Clinical Interview (SCID)	50 patients	29 (58%) patients received diagnosis disclosure
Kim, M.K. and Alvi, A. (1999) [2]	Otolaryngology—Head and Neck Surgery Department, at Temple University School of Medicine, Philadelphia, Pennsylvania, United States	Retrospective: evaluation through application of a self-developed questionnaire	16 patients	All patients received diagnosis disclosure
Lobb, E.A. et al. (2011) [20]	Tertiary referral centre for neurological cancers, Australia	Retrospective: evaluation through application of an original semi-structured interview	19 patients and 21 caregivers	All patients received diagnosis disclosure
Magro, E. et al. (2016) [21]	Department of Neurosurgery, University of Brest, Brest, France	Prospective: evaluation through application of an original self-developed satisfaction survey	91 patients	All patients received diagnosis disclosure
Malmstrom, A. et al. (2020) [22]	Neurosurgery, Oncology or Neurology Department, Linköping University, Sweden	Retrospective: evaluation through application of semi-structured interviews and the European Organization for Research and Treatment of Cancer (EORTC) QLQ-C30 quality of life (QoL) questionnaire with Brain Cancer Module BN20 and the MOCA test	25 patients	All patients received diagnosis disclosure
Motlagh, A. et al. (2014) [12]	11 major Iranian cancer centers, Iran	Retrospective: evaluation through application of an original self-developed questionnaire	82 patients	At the time of diagnosis, 34 (41.5%) patients received information disclosure. At the time of the final interview, 59 (72%) were aware of their diagnosis
Nwankwo, K.C. et al. (2013) [23]	Oncology Center, Nigeria Teaching Hospital, University of Nigeria, Enugu, Nigeria	Retrospective: evaluation through application of a questionnaire (modified by Yun et al.)	17 patients	10 (58.8%) patients received diagnosis disclosure
Perera M.C. et al. (2013) [24]	Otorhinolaryngology and Head and Neck Clinic, Teaching Hospital, Anuradhapura, Sri Lanka	Retrospective: evaluation through application of an original self-developed questionnaire	31 patients, 22 relatives, and 36 physicians	NI
Salander, P. and Spetz, A. (2002) [25]	Regional Hospital, Umea University, Umea, Sweden	Prospective: evaluation through three interviews for cognitive and emotional assessment through the Standardized Mini-Mental State Examination (SMMSE) and the Reaction to Diagnosis of Cancer questionnaire (RDCQ)	25 patients and 24 spouses	All patients received diagnosis disclosure
Umeda, M. et al. (2003) [5]	Graduate School of Medicine, Kobe University, Japan	Retrospective: evaluation through application of an original questionnaire	56 patients	50 (89%) patients received diagnosis disclosure
Wang, D.C. et al. (2011) [10]	Department of Oral and Maxillofacial Surgery, School and Hospital of Peking University, Beijing, China	Retrospective: evaluation through application of an original self-developed questionnaire and semi-structured interview	151 patients and 151 relatives	97 (64.2%) patients received diagnosis disclosure
Yamamoto, F. et al. (2011) [26]	Graduate School of Medicine, Osaka University, Japan	Retrospective: evaluation through application of an original questionnaire (modified by Narita et al.)	141 physicians	NA

NA: Not applicable; NI: No information. * The number of patients that the assessed relatives took care of was recorded, even though they were not evaluated.

**Table 2:** Aims, major findings, and key points from the included studies.

Author (Year)	Aim	Major Findings	Conclusions
Burton, M.V. and Parker, R.W. (1997) [11]	To study UK surgeons’ accounts of their practice regarding psychological aspects of cancer surgery, psychiatric morbidity, difficult patients, and care of the dying patients.	Five (62.5%) HN surgeons consistently disclose the presence of malignancy to their patients, while 2 (25%) make this decision on the patient’s or their relatives’ preferences, and another (12.5%) primarily tells exclusively the relatives. Less commonly discussed topics among patients and physicians include cause of the disease and the effects of surgery.	Respondents generally accept and respect patients’ wishes regarding truth disclosure. They struggle with their role as giver of bad news and with the consequent emotional reactions of the patient, pointing to the need for training and support of these professionals.
Costantini, M. et al. (2006) [17]	To estimate the proportion of Italian patients who died of cancer and that had been informed about diagnosis and prognosis, and to explore the variables associated with disclosure.	In total, 470 (37%) individuals who died from cancer received diagnostic disclosure. Regarding HNC patients, 21 (74%) received information disclosure. A consistent proportion of patients were aware of their disease without receiving formal information. Patients who died from head and neck cancer (OR = 4.7; 95% CI 1.2–19.3) had higher odds of being informed compared to referents. Higher education levels and a longer diagnosis-to-death interval significantly increased the patients’ probability of being informed, while advancing age significantly decreased this likelihood (*p* < 0.001). No significant relationship was found with the type of caregiver (*p* = 0.34), which were mostly female relatives (spouse or child).	In Italy, the practice of withholding the truth from cancer patients remains common among physicians. Recent cultural shifts toward a less paternalistic approach in medical care may not be significant in clinical settings.
Goebel, S. and Mehdorn, H.M. (2018) [18]	To assess the perspective of patients with intracranial tumors regarding the content of bad news, communication preferences, and clinical consequences of mismatch of patients’ communication preferences.	Twenty-eight patients (54%) met scores that described clinically relevant levels of cancer-related distress. Nine patients (14%) reported high levels of HADS anxiety and four (7%) of HADS depression. Patients with a more malignant tumor classification reported more communication needs (*p* = 0.609) and a higher need for information (*p* = 0.501). On average, 30% of patients’ preferences were not matched with the physicians’ behavior. Communication mismatch was associated with lower patient satisfaction regarding information but no other areas of psychosocial well-being.	Communicating bad news to the patient is a demanding endeavor for the treating physician that requires communication skills and accounting for the specific needs ascribable to the neurologic features of the disease (e.g., regarding neuropsychological impairment or neurosurgical treatment). Both content and preferences of bad news are often highly individual and specific for patients with brain tumors.
Graner, K.M. et al. (2015) [19]	To describe the sociodemographic characteristics, perceptions, expectations, and psychological symptoms of patients during the process of oral cancer diagnosis.	Twenty (69%) patients understood their diagnosis as cancer, while nine (31%) lacked understanding of the information provided, such that the participant’s response made no mention of their diagnosis. When faced with the diagnosis, 17 (58.6%) HNC patients experienced negative feelings, such as concern, nervousness, sadness, and anger. A higher prevalence (83.3%) of depressive symptoms was observed among those who received a diagnosis of cancer (*p* = 0.02), with an overall prevalence of 36.7%.	Professional support and care for the patient’s psychological state during the diagnosis process is crucial to enhance patient adherence and improve prognosis.
Hosaka, T. et al. (1999) [6]	To examine the prevalence rate of psychiatric disorders and the effects of full disclosure in two samples of otolaryngology patients (50 with malignant conditions and 50 patients with benign conditions).	Twenty-nine (58%) patients with cancer were not informed of their true condition. Twenty-nine (58%) family members who had relatives with cancer opted to withhold the information about his/her condition. Twenty-three (46%) patients with malignant diseases experienced psychiatric disorders. The total prevalence rate of psychiatric disorders in the informed (42.9%, 9 out of 21) and uninformed (48.3%, 14 out of 29) groups with malignant diseases showed no significant differences (χ^2^ = 0.144, df = 1, *p* = 0.704). The overall comparison between the groups of patients with benign and malignant diseases was not statistically significant (χ^2^ = 7.1, df = 1, *p* = 0.008), but depression was more frequently observed among malignant cancer patients (*p* < 0.05).	It is suggested that diagnostic concealment was not related to the presence of psychiatric disorders in this sample.
Kim, M.K. and Alvi, A. (1999) [2]	To evaluate the thoughts and concerns of patients receiving a diagnosis of head and neck cancer.	Thirteen (81.25%) patients received their diagnosis directly from the physician, while three (18.75%) became aware of it through friends and family. Thirteen (80%) felt that the cancer disclosure happened at a convenient time without interruptions. Fifteen (94%) patients were satisfied with the content of the information, did not require further clarification, and found the physician truthful and patient. Additionally, 81% reported that the physician’s presence was the most helpful aspect, and 82% did not wish to have anyone else present during the diagnosis. Following the disclosure, 75% of the patients experienced sadness, while 25% expressed anger.	Breaking bad news was a difficult and challenging task for most physicians. Patients want their HCN diagnosis delivery in simple and direct terms, and want their physician to be truthful, caring, and compassionate.
Lobb, E.A. et al. (2011) [20]	To seek the views of patients and their caregivers’ perceptions of the initial communication about the diagnosis of high-grade glioma and its prognosis.	All interviewed patients (n = 19) and caregivers (n = 21) expressed shock and disbelief upon learning about the diagnosis. Only 2 out of 19 patients and 21 caregivers reported a positive experience on the communication skills of staff when first given the diagnosis.	Effective communication regarding prognosis is crucial, enabling patients and their partners to confront the gravity of the situation openly. The study suggests that in-depth prognosis discussions should involve senior medical staff or advanced trainees with communication training and proven skills, and recommends clinicians to assess patient preferences for information preferences and tailor discussions accordingly.
Magro, E. et al. (2016) [21]	(a) To assess the implementation of the disclosure process team as mandated by the French cancer plans in a neurosurgical unit, (b) to characterize the impact of the disclosure process on the overall care of patients, and (c) to describe challenges and elements amenable to change.	On average, medical disclosure with the neurosurgeon occurred 11 days after surgery. Twenty-six (28.6%) patients were monitored by a psychologist or social worker, twenty-five (27.4%) connected with the oncology network, and four (4.4%) engaged with cancer communities. Forty-three patients (47.2%) of the total sample responded to the questionnaire. Initial information regarding the disclosure process was given by the neurosurgeon in 23 (53.4%) cases, and by a nurse in 16 (37.2%) cases. During the neurosurgeon consultation, 37 (86%) patients reported to receive information about diagnosis and disease and 33 (76.7%) about potential treatments with side effects. In 35 (81.4%) cases, patients found the time spent with the neurosurgeon adequate, and 18 (41.9%) preferred written information in addition to verbal information. The timing of the visit was considered right in 31 (72%) cases. After learning about the disclosure process, 19 (44.2%) patients felt reassured, 14 (32.5%) were surprised, and 6 (14%) were anxious.	Patients were generally satisfied with the quality of the disclosure process regarding information given, psychological support, and communication with all healthcare providers. It is suggested to provide early and patient-tailored psychological support coordinated by disclosure process nurses before physician disclosure of the diagnosis, to anticipate the needs and concerns of patients and their families regarding quality of life with a disclosure visit, and to use the time when the patient is unaware of the diagnosis to prepare the patient for the difficult moment of diagnosis disclosure.
Malmstrom, A. et al. (2020) [22]	To explore glioma patients’ experiences and preferences regarding receiving information on diagnosis and prognosis.	Participants generally wanted to know “the truth” about diagnosis and prognosis, but what they meant by “the truth” and how it should be communicated varied. Information on diagnosis was most often received directly from a physician at a personal meeting, often causing shock. Disclosure experience was categorized as either indirect (unplanned, causing fear and anxiety), insufficiently tailored (lacking in many aspects), or individualized and compassionate. Patients reported negative experiences when information was not adapted to their needs, preferences, and timing, and when it contained too little or too much detail. Participants’ MOCA scores were normal for 15 (60%) individuals, while 10 (40%) showed lower scores indicating cognitive impairment. Quality of life (QoL) data revealed fatigue and drowsiness as significant concerns, with role functioning being most affected.	To achieve patient-centered consultations, information on disease and prognosis, but also on practical issues, needs to be adapted to each patient regarding amount, detail, and timing, since patients have different individual preferences.
Motlagh, A. et al. (2014) [12]	To evaluate the preference of cancer patients for knowing the truth about their disease, as well as the factors that might have an impact on these preferences.	From the HNC group, 62 (75.6%) patients received information about their cancer diagnosis primarily from physicians (*p* = 0.05), 8 (9.7%) by professional caregivers or relatives, 4 (4.9%) from other sources, such as fellow patients, and 8 (9.7%) received information by unknown origin. Also, 69 (84.1%) patients were willing to receive more information about their disease. Thirty-three (40.2%) patients referred to their disease using the term “mass”, thirty (36.6%) used the term “cancer”, six (7.3%) used the term “disease”, three (3.6%) used the term “injury”, seven (8.5%) used other terms, and two (2.4%) used multiple terms. Patient preferences for decision-making were physician-led in 45 cases (54.8%), and their awareness of the malignancy of their disease at diagnosis was associated with having head and neck cancer (*p* < 0.001). Patients with brain tumors more frequently preferred shared decision-making with their physician.	The majority of Iranian cancer patients express a preference for being informed about the nature and prognosis of their cancer, with many willing to play an active role in treatment decision-making. Understanding the factors influencing this preference may help categorize patients based on their desired level of information.
Nwankwo, K.C. et al. (2013) [23]	To ascertain disclosure information and needs from cancer patients from southeast Nigeria.	In total, 10 (62.5%) HNC patients did not request for diagnosis information. Half of the HNC patients reported that the explanation of their sickness was adequate enough.	The findings underscore the importance of considering individual patient preferences in disclosing cancer diagnoses, suggesting that physicians in southeast Nigeria should tailor their practices to meet these diverse information needs.
Perera M.C. et al. (2013) [24]	To study the attitudes of doctors, cancer patients, and their close family members about informing the diagnosis of HNC.	Twenty-nine (93.6%) patients, twelve relatives (45.5%), and twenty-one (58.3%) physicians wanted the cancer diagnosis to be disclosed. Twelve (54.6%) relatives and fifteen (41.7%) doctors felt it was best to inform the relatives before the patient, but only two (6.4%) patients agreed to this claim and sixteen (45.7%) doctors said they would accede to the family’s request not to tell the patient the cancer diagnosis. Thirty-eight (71.7%) patients and their relatives wanted the word “cancer” to be used, while only nineteen (52.8%) doctors adhered to this practice. Seventy-nine (88.7%) patients wanted the doctor to disclose the information, while eight (8.9%) wanted a family member to do it. Out of the doctors, 22 (62.8%) were comfortable in discussing the diagnosis of cancer. Forty (75%) patients and their family members wanted the information of cancer to be given to them in the first visit, while twenty-one (60%) doctors preferred to tell them gradually using many visits.	It is suggested that HNC patients in Anuradhapura have no inhibition of accepting their diagnosis of cancer and its complications.
Salander, P. and Spetz, A. (2002) [25]	To contribute to knowledge of how couples communicate regarding the fact that a family member is dying of cancer.	Four distinct social processes were detected in relation to family communication about a cancer diagnosis: (1) the patient does not seem to be aware, the spouse is aware but pretends not to be; (2) both are aware, but the patient does not want to share—they drift apart; (3) both are aware, they do/do not talk openly about the gravity of the situation; nevertheless, there is a joint platform; (4) neither patient nor spouse seems to be aware, and they carry on living as before.The patients, compared to the spouses, seemed content with the received information. Patients who shared a mutual understanding with their spouses formed a “joint platform” to navigate their impending challenges. However, it is common for patient–spouse couples to conceal rather than reveal the terminal aspects of the disease from each other.	In about half of the cases, patients with brain tumors and their spouses did not openly share critical information about the situation. The patients’ reluctance to engage in dialogue was mainly attributed to cognitive deficiencies and personality traits. In the remaining half, a subtle mutual acknowledgment, rather than open awareness, appeared significant. In these instances, the situation could be characterized as living despite the awareness of dying.
Umeda, M. et al. (2003) [5]	To examine, using a questionnaire, the requests of patients with oral cancer for disclosure of diagnosis, self-choice of treatment, and second opinion, and to discuss the proper method for disclosure of diagnosis to Japanese cancer patients.	Fifty (89%) expressed a desire for accurate information about their illness, irrespective of its nature. No psychological consequences were reported as a result of the disclosure. For the other 6 (11%) patients who asked for collusion, the term “disease” was used by the doctors. Five (9%) patients said they wanted a second opinion. Forty-three (77%) patients preferred to leave treatment decisions to their doctors. There was no observed correlation between responses to questions and patients’ age or sex.	Most patients hope to receive information about their diagnosis.
Wang, D.C. et al. (2011) [10]	To study cancer patients’ awareness of their diagnosis and to determine who tends to disclose bad news to cancer patients.	Twenty (20.6%) of the aware patients were informed by physicians, seventeen (17.5%) were informed by relatives, and sixty (61.9%) obtained the information on their own (i.e., access to medical records or changes in their relatives’ behavior). Patients with a higher level of education were less likely to have had their cancer diagnosis concealed from them. No association was noted between diagnosis awareness and the patients’ age, gender, cancer type, disease stage, hospital, or residential area.	Despite efforts by family members to achieve diagnosis concealment, a significant number of patients in China discovered their oral and maxillofacial cancer diagnosis on their own. This could indicate that therapeutic non-disclosure is not highly effective in concealing the truth.
Yamamoto, F. et al. (2011) [26]	To determine the current status of disclosure to glioma patients in Japan and to analyze the factors associated with disclosure.	Physicians disclose diagnosis to glioblastoma patients aged < 60 years 44.3% of the time, and 41.4% for those aged > 70 years; for anaplastic astrocytoma patients, these proportions were 61.5% and 51.9%, respectively. Factors increasing disclosure frequency included physicians working at facilities performing over 50 glioma cases per year, those in metropolitan areas, and those with additional psychosocial support systems for patients. When families opposed disclosure, over half of the physicians respected their wishes. The physicians’ gender and postgraduate practice period did not affect disclosure practices.	Physicians generally informed patients with malignant glioma about the malignant nature of the disease, but often withheld the exact diagnosis. Their disclosure practices varied based on factors such as histopathological grading, hospital case volume, location, availability of patient psychological support systems, and family wishes. The need for patients’ support from other healthcare professionals besides the surgical neuro-oncologists is highlighted.

Author (Year)

Aim

Major Findings

Conclusions

## Data Availability

Data supporting the findings of this study are available in the Supplementary Materials and from the corresponding author upon reasonable request.

## Supplementary Materials

The following supporting information can be downloaded at: https://www.mdpi.com/article/10.3390/dj12070214/s1. Online Resource S1: Preferred Reporting Items for Systematic Reviews and Meta-Analyses extension for Scoping Reviews (PRISMA-ScR) Checklist. Online Resource S2: Search strategy. Online Resource S3: Sociodemographic and clinical characteristics of the study population. Online Resource S4: Risk-of-bias assessment according to the Joanna Briggs Institute Critical Appraisal Tool for each study design. (A) Cross-sectional studies, (B) case-control studies, and (C) cohort studies. Online Resource S5: Risk-of-bias assessment checklists according to the Joanna Briggs Institute Critical Appraisal Tool for each study [2,5,6,10,11,12,17,18,19,20,21,22,23,24,25,26].

## References

[1] Costa V., Da Silva E., Maria M., Zago F. (2005). A Revelação Do Diagnóstico de Câncer Para Profissionais e Pacientes. Rev. Bras. Enferm..

[2] Kim M.K., Alvi A. (1999). Breaking the Bad News of Cancer: The Patient’s Perspective. Laryngoscope.

[3] Martins B.N.F.L., Migliorati C.A., Ribeiro A.C.P., Martins M.D., Brandão T.B., Lopes M.A., Alves C.G.B., Santos-Silva A.R. (2023). The Barriers Dentists Face to Communicate Cancer Diagnosis: Self-Assessment Based on SPIKES Protocol. Med. Oral Patol. Oral Cir. Bucal..

[4] Alves C.G.B., Treister N.S., Ribeiro A.C.P., Brandão T.B., Tonaki J.O., Lopes M.A., Rivera C., Santos-Silva A.R. (2020). Strategies for Communicating Oral and Oropharyngeal Cancer Diagnosis: Why Talk about It?. Oral Surg. Oral Med. Oral Pathol. Oral Radiol..

[5] Umeda M., Komatsubara H., Minamikawa T., Furudoi S., Shibuya Y., Yokoo S., Komori T. (2003). A Questionnaire on Requests for Disclosure of Diagnosis, Self-Choice of Treatment, and Second Opinion of Patients with Oral Cancer in Japan. J. Palliat. Care.

[6] Hosaka T., Awazu H., Fukunishi I., Okuyama T., Wogan J. (1999). Disclosure of True Diagnosis in Japanese Cancer Patients. Gen. Hosp. Psychiatry.

[7] Juneja H., Aggarwal P., McCord C. (2021). Impact of the COVID-19 Pandemic on the Diagnosis of Oral and Maxillofacial Malignancies: A Retrospective Study. J. Can. Dent. Assoc..

[8] Sutar R., Chaudhary P., Yadav V. (2022). Prevalence of Collusion in Cancer Communications: A Meta-Analysis. Psychooncology.

[9] Pulcini R., D’agostino S., Dolci M., Bissioli A., Caporaso L., Iarussi F. (2022). The Impact of COVID-19 on Oral Cancer Diagnosis: A Systematic Review. J. Multidiscip. Appl. Nat. Sci..

[10] Wang D.C., Bin Guo C., Peng X., Su Y.J., Chen F. (2011). Is Therapeutic Non-Disclosure Still Possible? A Study on the Awareness of Cancer Diagnosis in China. Support. Care Cancer.

[11] Burton M.V., Parker R.W. (1997). Psychological Aspects of Cancer Surgery: Surgeons’ Attitudes and Opinions. Psychooncology.

[12] Motlagh A., Mafi A.R., Yaseri M., Hemati S. (2014). Attitude of Cancer Patients toward Diagnosis Disclosure and Their Preference for Clinical Decision-Making: A National Survey. Arch. Iran. Med..

[13] Montazeri A., Tavoli A., Mohagheghi M.A., Roshan R., Tavoli Z. (2009). Disclosure of Cancer Diagnosis and Quality of Life in Cancer Patients: Should It Be the Same Everywhere?. BMC Cancer.

[14] Tricco A.C., Lillie E., Zarin W., O’Brien K.K., Colquhoun H., Levac D., Moher D., Peters M.D.J., Horsley T., Weeks L. (2018). PRISMA Extension for Scoping Reviews (PRISMA-ScR): Checklist and Explanation. Ann. Intern. Med..

[15] Peters M.D.J., Godfrey C., McInerney P., Munn Z., Tricco A.C., Khalil H. (2020). Chapter 11: Scoping Reviews (2020 Version). JBI Manual for Evidence Synthesis.

[16] Page M.J., McKenzie J.E., Bossuyt P.M., Boutron I., Hoffmann T.C., Mulrow C.D., Shamseer L., Tetzlaff J.M., Akl E.A., Brennan S.E. (2021). The PRISMA 2020 statement: An updated guideline for reporting systematic reviews. BMJ.

[17] Costantini M., Morasso G., Montella M., Borgia P., Cecioni R., Beccaro M., Sguazzotti E., Bruzzi P., Sormani M.P., Merlo F. (2006). Diagnosis and Prognosis Disclosure among Cancer Patients. Results from an Italian Mortality Follow-Back Survey. Ann. Oncol..

[18] Goebel S., Mehdorn H.M. (2018). Breaking Bad News to Patients with Intracranial Tumors: The Patients’ Perspective. World Neurosurg..

[19] Graner K.M., Rolim G.S., Moraes A.B.A., Padovani C.R., Lopes M.A., Santos-Silva A.R., Ramos-Cerqueira A.T.A. (2016). Feelings, perceptions, and expectations of patients during the process of oral cancer diagnosis. Support Care Cancer..

[20] Lobb E.A., Halkett G.K.B., Nowak A.K. (2011). Patient and Caregiver Perceptions of Communication of Prognosis in High Grade Glioma. J. Neurooncol..

[21] Magro E., Bergot L., Cuchard S., Lebreton S., Coutte M.B., Rolland-Lozachmeur G., Hieu P.D., Seizeur R. (2016). Diagnosis disclosure process in patients with malignant brain tumors. Clin. J. Oncol. Nurs..

[22] Malmström A., Åkesson L., Milos P., Mudaisi M., Bruhn H., Strandeus M., Karlsson M. (2021). “Do I want to know it all?” A qualitative study of glioma patients’ perspectives on receiving information about their diagnosis and prognosis. Support Care Cancer..

[23] Nwankwo K.C., Anarado A.N., Ezeome E.R. (2013). Attitudes of cancer patients in a university teaching hospital in southeast Nigeria on disclosure of cancer information. Psycho-Oncol..

[24] Perera M., Tennakoon T., Kumarasiri L., Jayasinghe S., Rathnayake R., Rajapaksha R. (2013). Cancer in Sri Lanka: The question of, “to tell or Not to tell”. Ceylon J. Otolaryngol..

[25] Salander P., Spetz A. (2002). How do patients and spouses deal with the serious facts of malignant glioma?. Palliat Med..

[26] Yamamoto F., Hashimoto N., Kagawa N., Okita Y., Chiba Y., Kijima N., Kinoshita M., Yoshizu K., Fujimoto Y., Hirai K. (2011). A survey of disclosure of diagnosis to patients with glioma in Japan. Int. J. Clin. Oncol..

[27] Wang D.C., Bin Guo C., Peng X., Su Y.J. (2014). Psychological Morbidity and Health-Related Quality of Life in Patients with Differing Awareness of Cancer Diagnosis: A Cross-Sectional Study. Psychooncology.

[28] Khalil R.B. (2012). Attitudes, Beliefs and Perceptions Regarding Truth Disclosure of Cancer-Related Information in the Middle East: A Review. Palliat. Support. Care.

[29] Zamanzadeh V., Rahmani A., Valizadeh L., Ferguson C., Hassankhani H., Nikanfar A.R., Howard F. (2013). The Taboo of Cancer: The Experiences of Cancer Disclosure by Iranian Patients, Their Family Members and Physicians. Psychooncology.

[30] Arboleda L.P.A., Pereira T.C.E., Epstein J.B., Migliorati C.A., Warnakulasuriya S., Diniz-Freitas M., Lopes M.A., Santos-Silva A.R. (2023). Clinical and Psychosocial Impact of Oral Potentially Malignant Disorders Communication: A Scoping Review. Dent. J..

[31] Schaefer I., Heneka N., Luckett T., Agar M.R., Chambers S.K., Currow D.C., Halkett G., Disalvo D., Amgarth-Duff I., Anderiesz C. (2021). Quality of Online Self-Management Resources for Adults Living with Primary Brain Cancer, and Their Carers: A Systematic Environmental Scan. BMC Palliat. Care.

[32] Sutar R., Chaudhary P. (2022). Prognostic Disclosure in Cancer Care: A Systematic Literature Review. Palliat. Care Soc. Pract..

[33] Ghavamzadeh A., Bahar B. (1997). Communication with the cancer patient in Iran. Inf. Truth. Ann. N. Y. Acad. Sci..

[34] Feldman M.D., Zhang J., Cummings S.R. (1999). Chinese and U.S. Internists Adhere to Different Ethical Standards. J. Gen. Intern. Med..

[35] Chen S.H., Chen S.Y., Yang S.C., Chien R.N., Chen S.H., Chu T.P., Fujimori M., Tang W.R. (2021). Effectiveness of Communication Skill Training on Cancer Truth-Telling for Advanced Practice Nurses in Taiwan: A Pilot Study. Psychooncology.

[36] Tsoussis S., Papadogiorgaki M., Markodimitraki E., Delibaltadakis G., Strevinas A., Psyllakis M., Tabakaki K., Drossitis I., Kabourakis A., Papadimitraki E. (2013). Disclosure of Cancer Diagnosis: The Greek Experience. JBUON.

[37] Bruera E., Mazzocato C., Stiefel Centre Hopitalier Universitaire Vaudois F., Sala Hospital Eva Peron R. (2000). Attitudes and Beliefs of Palliative Care Physicians Regarding Communication with Terminally Ill Cancer Patients. Palliat. Med..

[38] Pinto R.N., Cristina Chaves A., Theresa Lourenço M., de Jesus Mari J. (2004). Information Needs of Recently Diagnosed Cancer Patients in Brazil. Psychiatry Med..

[39] Chittem M., Butow P. (2015). Responding to Family Requests for Nondisclosure: The Impact of Oncologists’ Cultural Background. J. Cancer Res. Ther..

[40] Innes S., Payne S. (2009). Advanced Cancer Patients’ Prognostic Information Preferences: A Review. Palliat. Med..

[41] Gaston C.M., Mitchell G. (2005). Information Giving and Decision-Making in Patients with Advanced Cancer: A Systematic Review. Soc. Sci. Med..

[42] Jiang Y., Li J.Y., Liu C., Huang M.J., Zhou L., Li M., Zhao X., Wei Y.Q. (2006). Different Attitudes of Oncology Clinicians toward Truth Telling of Different Stages of Cancer. Support. Care Cancer.

[43] Nie X., Ye D., Wang Q., Manyande A., Yang L., Qiu H., Chao T., Zhang P., Gong C., Zhuang L. (2016). Poor-Prognosis Disclosure Preference in Cancer Patient–Caregiver Dyads and Its Association with Their Quality of Life and Perceived Stress: A Cross-Sectional Survey in Mainland China. Psychooncology.

[44] Grassi L., Giraldi T., Messina E.G., Magnani K., Valle E., Cartei G. (2000). Physicians’ Attitudes to and Problems with Truth-Telling to Cancer Patients. Support. Care Cancer.

[45] Roscoe L.A., Tullis J.A., Reich R.R., McCaffrey J.C. (2013). Beyond Good Intentions and Patient Perceptions: Competing Definitions of Effective Communication in Head and Neck Cancer Care at the End of Life. Health Commun..

[46] Bloom J.R., Marshall D.C., Rodriguez-Russo C., Martin E., Jones J.A., Dharmarajan K.V. (2022). Prognostic Disclosure in Oncology—Current Communication Models: A Scoping Review. BMJ Support. Palliat. Care.

[47] Mahendiran M., Yeung H., Rossi S., Khosravani H., Perri G.A. (2023). Evaluating the Effectiveness of the SPIKES Model to Break Bad News—A Systematic Review. Am. J. Hosp. Palliat. Care.

[48] Lin C.-C. (1999). Disclosure of the Cancer Diagnosis as It Relates to the Quality of Pain Management Among Patients with Cancer Pain in Taiwan. J Pain Symptom Manag..

[49] Ghoshal A., Salins N., Damani A., Chowdhury J., Chitre A., Muckaden M.A., Muckaden A., Deodhar J., Badwe R. (2019). To Tell or Not to Tell: Exploring the Preferences and Attitudes of Patients and Family Caregivers on Disclosure of a Cancer-Related Diagnosis and Prognosis. J. Glob. Oncol..

[50] Bronheim H., Strain J.J., Biller H.F. (1991). Psychiatric Aspects of Head and Neck Surgery Part I: New Surgical Techniques and Psychiatric Consequences. Gen. Hosp. Psychiatry.

[51] Davies A.D.M., Daviesand C., Delpo M.C. (1986). Depression and Anxiety in Patients Undergoing Diagnostic Investigations for Head and Neck Cancers. Br. J. Psychiatry.

