# Supplementary material for: Conspiracy of Silence in Head and Neck Cancer Diagnosis: A Scoping Review
Source: Dent J (Basel). 2024 Jul 11;12(7):214. doi: 10.3390/dj12070214 (PMC11276277; doi:10.3390/dj12070214)
Supplement: Supplementary file 1 [file dentistry-12-00214-s001.zip › dentistry-3050680-supplementary.pdf]

Online Resource S1. Preferred Reporting Items for Systematic reviews and Meta-Analyses extension for Scoping Reviews (PRISMA-ScR) Checklist

Section	Item	PRISMA-ScR checklist item	Reported on page #
**TITLE**			
**Title**	1	Identify the report as a scoping review.	1
**ABSTRACT**			
**Structured summary**	2	Provide a structured summary that includes (as applicable): background, objectives, eligibility criteria, sources of evidence, charting methods, results, and conclusions that relate to the review questions and objectives.	1
**INTRODUCTION**			
**Rationale**	3	Describe the rationale for the review in the context of what is already known. Explain why the review questions/objectives lend themselves to a scoping review approach.	2
**Objectives**	4	Provide an explicit statement of the questions and objectives being addressed with reference to their key elements (e.g., population or participants, concepts, and context) or other relevant key elements used to conceptualize the review questions and/or objectives.	2, 3
**METHODS**			
**Protocol and registration**	5	Indicate whether a review protocol exists; state if and where it can be accessed (e.g., a Web address); and if available, provide registration information, including the registration number.	2
**Eligibility criteria**	6	Specify characteristics of the sources of evidence used as eligibility criteria (e.g., years considered, language, and publication status), and provide a rationale.	3
**Information sources***	7	Describe all information sources in the search (e.g., databases with dates of coverage and contact with authors to identify additional sources), as well as the date the most recent search was executed.	3
**Search**	8	Present the full electronic search strategy for at least 1 database, including any limits used, such that it could be repeated.	Online Resource S1

**Selection of sources of evidence†**	9	State the process for selecting sources of evidence (i.e., screening and eligibility) included in the scoping review.	4
**Data charting process‡**	10	Describe the methods of charting data from the included sources of evidence (e.g., calibrated forms or forms that have been tested by the team before their use, and whether data charting was done independently or in duplicate) and any processes for obtaining and confirming data from investigators.	4
**Data items**	11	List and define all variables for which data were sought and any assumptions and simplifications made.	4
**Critical appraisal of individual sources of evidence§**	12	If done, provide a rationale for conducting a critical appraisal of included sources of evidence; describe the methods used and how this information was used in any data synthesis (if appropriate).	4
**Synthesis of results**	13	Describe the methods of handling and summarizing the data that were charted.	6
**RESULTS**			
**Selection of sources of evidence**	14	Give numbers of sources of evidence screened, assessed for eligibility, and included in the review, with reasons for exclusions at each stage, ideally using a flow diagram.	5
**Characteristics of sources of evidence**	15	For each source of evidence, present characteristics for which data were charted and provide the citations.	16
**Critical appraisal within sources of evidence**	16	If done, present data on critical appraisal of included sources of evidence (see item 12).	18
**Results of individual sources of evidence**	17	For each included source of evidence, present the relevant data that were charted that relate to the review questions and objectives.	18
**Synthesis of results**	18	Summarize and/or present the charting results as they relate to the review questions and objectives.	Tables 1 and 2
**DISCUSSION**			
**Summary of evidence**	19	Summarize the main results (including an overview of concepts, themes, and types of evidence available), link to the review questions and objectives, and consider the relevance to key groups.	21

**Limitations**	20	Discuss the limitations of the scoping review process.	24
**Conclusions**	21	Provide a general interpretation of the results with respect to the review questions and objectives, as well as potential implications and/or next steps.	24
**FUNDING**			
**Funding**	22	Describe sources of funding for the included sources of evidence, as well as sources of funding for the scoping review. Describe the role of the funders of the scoping review.	25

*NA: Not applicable.

Online Resource S2. Search strategy

Database	Search strategy (Search date: December 14th, 2023)	Results
**PubMed**	((Cancer OR Malignancy OR "Malignant Neoplasm") AND ("truth disclosure"[MeSH Terms] OR "truth disclosure" OR nondisclosure OR "information disclosure" OR collusion OR "pact of silence" OR "pacto de silencio" OR "conspiracy of silence" OR "concealment" OR "patient autonomy model" OR "family-oriented model" OR "doctor-patient relationship" OR "medical paternalism" OR "right to information" OR truth telling OR "professional-patient communication" OR censorship OR paternalism OR "bad news"))	3,976
**Embase**	('cancer'/de OR 'malignancy'/de OR 'malignant neoplasm'/de) AND (('truth disclosure'/de OR nondisclosure OR 'information disclosure' OR collusion OR 'pact of silence' OR 'pacto de silencio' OR 'conspiracy of silence' OR 'concealment' OR 'patient autonomy model' OR 'family-oriented model' OR 'doctor-patient relationship'/de OR 'medical paternalism' OR 'right to information' OR 'truth'/de) AND telling OR 'professional-patient communication' OR 'censorship'/de OR 'paternalism'/de OR 'bad news')	124
**Web of Science**	TEMA: (Cancer OR Malignancy OR "Malignant Neoplasm") AND TEMA: ("truth disclosure" OR nondisclosure OR "information disclosure" OR collusion OR "pact of silence" OR "pacto de silencio" OR "conspiracy of silence" OR "concealment" OR "patient autonomy model" OR "family-oriented model" OR "doctor-patient relationship" OR "medical paternalism" OR "right to information" OR truth telling OR "professional-patient communication" OR censorship OR paternalism OR "bad news")	2,658
**LILACS**	cancer AND ("truth disclosure" OR nondisclosure OR "information disclosure" OR collusion OR "pact of silence" OR "pacto de silencio" OR "conspiracy of silence" OR "concealment" OR "patient autonomy model" OR "family-oriented model" OR "doctor-patient relationship" OR "medical paternalism" OR "right to information" OR truth telling OR "professional-patient communication" OR "bad news")	149
**SCOPUS**	TITLE-ABS-KEY(Cancer OR Malignancy OR "Malignant Neoplasm") AND TITLE-ABS-KEY("truth disclosure" OR nondisclosure OR "information disclosure" OR collusion OR "pact of silence" OR "pacto de silencio" OR "conspiracy of silence" OR "concealment" OR "patient autonomy model" OR "family oriented model" OR "doctor-patient relationship" OR "medical paternalism" OR "right to information" OR truth telling OR "professional-patient communication" OR censorship OR paternalism OR "bad news")	732
	**Grey Literature**	
**Google Scholar**	(Cancer OR Malignancy) AND ("truth disclosure" OR nondisclosure OR "information disclosure" OR collusion OR "pact of silence" OR "pacto de silencio" OR "conspiracy of silence" OR "concealment")	200
**ProQuest**	TI,AB(Cancer OR Malignancy OR "Malignant Neoplasm") AND TI,AB("truth disclosure" OR nondisclosure OR "information disclosure" OR collusion OR "pact of silence" OR "pacto de silencio" OR "conspiracy of silence" OR "concealment" OR "patient autonomy model" OR "family-oriented model" OR "doctor-patient relationship" OR "medical paternalism" OR "right to information" OR truth telling OR "professional-patient communication" OR censorship OR paternalism OR "bad news")	1,399
**TOTAL**		9,238

Online Resource S3. Sociodemographic and clinical characteristics of study population (n=662)

Author (year)	Patients (n)	Mean age (range)	Sex distribution (F-M)	Type of tumor and location site
**Burton, M. V. and Parker, R. W (1997) [11]**	NA	NA	NA	NA
**Costantini, M. *et al* (2006) [16]**	28	NI	NI	Head and neck cancer (n=28)
**Goebel, S. and Mehdorn, H. M (2018) [17]**	42	51.31 (20-73)	17F-25M	Glioblastoma multiforme (n=12), lymphoma (n=4), meningioma (n=13), other (n=13)
**Graner, K. M. *et al* (2015) [18]**	29	NI	NI	Oral cancer (n=29)
**Hosaka, T. *et al* (1999) [6]**	50	55.9 (NI)	9F-41M	Pharyngeal cancer (n=23), laryngeal cancer (n=11), tongue cancer (n=7), lymphoma (n=4), maxillary cancer (n=3), other (n=2)
**Kim, M. K. and Alvi, A (1999) [2]**	16	63 (NI)	6F-10M	Laryngeal cancer (n=6), oropharyngeal cancer (n=5), hypopharyngeal cancer (n=2), oral cancer (n=2), nasopharyngeal carcinoma (n=1)
**Lobb, E. A. *et al* (2011) [19]**	19	NI	7F-12M	Glioblastoma multiforme grade IV (n=16), anaplastic gemistocystic astrocytoma grade III (n=2), astrocytoma grade IV (n=1)
**Magro, E. *et al* (2016) [20]**	91	59 (29-85)	35F-56M	Malignant brain tumor (n=91)
**Malmstrom, A. *et al* (2020) [21]**	25	51 (25-76)	10F-15M	Glioblastoma grade IV (n=12), oligodendroglioma grade II (n=9), astrocytoma grade II (n=3), anaplastic astrocytoma grade III (n=1)
**Motlagh, A. *et al* (2014) [12]**	82	NI	NI	Brain tumor (n=44), head and neck tumor (n=38)
**Nwankwo, K. C. *et al* (2013) [22]**	17	NI	NI	Head and neck cancer (n=17)
**Perera M.C. *et al* (2013) [23]**	31	NI	NI	Head and neck cancer (n=31)
**Salander, P. and Spetz, A (2002) [24]**	25	53.6 (32-70)	9F-16M	Malignant glioma grade III-IV(n=25)
**Umeda, M. *et al* (2003) [5]**	56	63.4 (31-83)	18-F38M	Oral cancer: tongue (n=24), mandible (n=11), maxilla (n=7), floor of mouth (n=5), buccal mucosa (n=4), palate (n=2), other (n=3)
**Wang, D.C. *et al* (2011) [10]**	151	56 (NI)	64F-87M	Squamous cell carcinoma (n=104), salivary gland malignant carcinoma (n=30), other (n=17)
**Yamamoto, F. *et al* (2011) [25]**	NA	43.2 (28-75)*	6F-135M*	NA

*Demographic information from the physicians involved.

Online Resource S4. Risk of bias assessment according to Joanna Brings Institute critical appraisal tool for each study design: (A) Cross-sectional studies; (B) Case-control studies; (C) Cohort studies.

(a) Cross-sectional studies: [2, 5, 10-12, 17-25]

Cross-sectional Studies (n=14)	Q1	Q2	Q3	Q4	Q5	Q6	Q7	Q8	BIAS RISK	
Yamamoto, F. *et al* (2011)									71.4%	Low
Wang, D.C. *et al* (2011)									71.4%	Low
Umeda, M. *et al* (2003)									14.4%	High
Salander, P. and Spetz, A (2002)									28.5%	High
Perera M.C. *et al* (2013)									0%	High
Nwankwo, K. C. *et al* (2013)									57.1%	Moderate
Motlagh, A. *et al* (2014)									57.1%	Moderate
Malmstrom, A. *et al* (2020)									62.5%	Moderate
Magro, E. *et al* (2016)									42.8%	High
Lobb, E. A. *et al* (2011)									42.8%	High
Kim, M. K. and Alvi, A (1999)									28.5%	High
Graner, K. M. *et al* (2015)									71.4%	Low
Goebel, S. and Mehdorn, H. M (2018)									57.1%	Moderate
Burton, M. V. and Parker, R. W (1997)									16.6%	High

Q1: Were the criteria for inclusion in the sample clearly defined? – Q2: Were the study subjects and the setting described in detail? – Q3: Was the exposure measured in a valid and reliable way? – Q4: Were objective, standard criteria used for measurement of the condition? – Q5: Were confounding factors identified? – Q6: Were strategies to deal with confounding factors stated? – Q7: Were the outcomes measured in a valid and reliable way? – Q8: Was appropriate statistical analysis used?

(b) Case-control studies: [6]

Case-control Studies (n=1)	Q1	Q2	Q3	Q4	Q5	Q6	Q7	Q8	Q9	Q10	BIAS RISK	
Hosaka, T. *et al* (1999)											75%	Low

Q1: Were the groups comparable other than the presence of disease in cases or the absence of disease in controls? – Q2: Were cases and controls matched appropriately? – Q3: Were the same criteria used for identification of cases and controls? – Q4: Was exposure measured in a standard, valid and reliable way? – Q5: Was exposure measured in the same way for cases and controls? – Q6: Were confounding factors identified? – Q7: Were strategies to deal with confounding factors stated? – Q8: Were outcomes assessed in a standard, valid and reliable way for cases and controls? – Q9: Was the exposure period of interest long enough to be meaningful? – Q10: Was appropriate statistical analysis used?

(c) Cohort studies: [16]

Cohort Studies (n=1)	Q1	Q2	Q3	Q4	Q5	Q6	Q7	Q8	Q9	Q10	Q11	BIAS RISK	
Costantini, M. *et al* (2006)												71.4%	Low

Q1: Were the two groups similar and recruited from the same population? – Q2: Were the exposures measured similarly to assign people to both exposed and unexposed groups? – Q3: Was the exposure measured in a valid and reliable way? – Q4: Were confounding factors identified? – Q5: Were strategies to deal with confounding factors stated? – Q6: Were the groups/participants free of the outcome at the start of the study (or at the moment of exposure)? – Q7: Were the outcomes measured in a valid and reliable way? – Q8: Was the follow up time reported and sufficient to be long enough for outcomes to occur? – Q9: Was follow up complete, and if not, were the reasons to loss to follow up described and explored? – Q10: Were strategies to address incomplete follow up utilized? – Q11: Was appropriate statistical analysis used?

© JBI, 2020. All rights reserved. JBI grants use of these tools for research purposes only. All other enquiries should be sent to jbisynthesis@adelaide.edu.au.

Online Resource S5. Risk of bias assessment checklists according to Joanna Brings Institute critical appraisal tool for each study.

JBI CRITICAL APPRAISAL CHECKLIST FOR ANALYTICAL CROSS SECTIONAL STUDIES

Reviewer: CSS and ESS Date: 24-06-2024

Authors: Burton, M. V. and Parker, R. W Year: 1997 Record Number: 1

	Yes	No	Unclear	Not applicable
1. Were the criteria for inclusion in the sample clearly defined?	☐	+	☐	☐
2. Were the study subjects and the setting described in detail?	☐	+	☐	☐
3. Was the exposure measured in a valid and reliable way?	+	☐	☐	☐
4. Were objective, standard criteria used for measurement of the condition?	☐	+	☐	☐
5. Were confounding factors identified?	☐	+	☐	☐
6. Were strategies to deal with confounding factors stated?	☐	☐	☐	+
7. Were the outcomes measured in a valid and reliable way?	☐	+	☐	☐
8. Was appropriate statistical analysis used?	☐	☐	☐	+

Overall appraisal: Include + Exclude ☐ Seek further info ☐

Comments (Including reason for exclusion)

© JBI, 2020. All rights reserved. JBI grants use of these tools for research purposes only. All other enquiries should be sent to jbisynthesis@adelaide.edu.au.

JBI CRITICAL APPRAISAL CHECKLIST FOR COHORT STUDIES

Reviewer: CSS and ESS Date: 24-06-2024

Author: Costantini, M. et al Year 2006 Record Number2

	Yes	No	Unclear	Not applicable
1. Were the two groups similar and recruited from the same population?	☐	☐	☐	+
2. Were the exposures measured similarly to assign people to both exposed and unexposed groups?	☐	☐	☐	+
3. Was the exposure measured in a valid and reliable way?	+	☐	☐	☐
4. Were confounding factors identified?	☐	☐	+	☐
5. Were strategies to deal with confounding factors stated?	☐	☐	☐	+
6. Were the groups/participants free of the outcome at the start of the study (or at the moment of exposure)?	☐	+	☐	☐
7. Were the outcomes measured in a valid and reliable way?	+	☐	☐	☐
8. Was the follow up time reported and sufficient to be long enough for outcomes to occur?	+	☐	☐	☐
9. Was follow up complete, and if not, were the reasons to loss to follow up described and explored?	+	☐	☐	☐
10. Were strategies to address incomplete follow up utilized?	☐	☐	☐	+
11. Was appropriate statistical analysis used?	+	☐	☐	☐

Overall appraisal: Include + Exclude ☐ Seek further info ☐

Comments (Including reason for exclusion)

JBI CRITICAL APPRAISAL CHECKLIST FOR ANALYTICAL CROSS SECTIONAL STUDIES

Reviewer: CSS and ESS Date: 24-06-2024

Author: Goebel, S. and Mehdorn, H. M Year: 2018 Record Number: 3

	Yes	No	Unclear	Not applicable
1. Were the criteria for inclusion in the sample clearly defined?	+	☐	☐	☐
2. Were the study subjects and the setting described in detail?	☐	+	☐	☐
3. Was the exposure measured in a valid and reliable way?	+	☐	☐	☐
4. Were objective, standard criteria used for measurement of the condition?	☐	+	☐	☐
5. Were confounding factors identified?	☐	+	☐	☐
6. Were strategies to deal with confounding factors stated?	☐	☐	☐	+
7. Were the outcomes measured in a valid and reliable way?	+	☐	☐	☐
8. Was appropriate statistical analysis used?	+	☐	☐	☐

Overall appraisal: Include + Exclude ☐ Seek further info ☐

Comments (Including reason for exclusion)

© JBI, 2020. All rights reserved. JBI grants use of these tools for research purposes only. All other enquiries should be sent to jbisynthesis@adelaide.edu.au.

JBI CRITICAL APPRAISAL CHECKLIST FOR ANALYTICAL CROSS SECTIONAL STUDIES

Reviewer: CSS and ESS Date: 24-06-2024

Author: Graner, K. M. et al Year:2015 Record Number: 4

	Yes	No	Unclear	Not applicable
1. Were the criteria for inclusion in the sample clearly defined?	+	☐	☐	☐
2. Were the study subjects and the setting described in detail?	+	☐	☐	☐
3. Was the exposure measured in a valid and reliable way?	+	☐	☐	☐
4. Were objective, standard criteria used for measurement of the condition?	☐	+	☐	☐
5. Were confounding factors identified?	☐	+	☐	☐
6. Were strategies to deal with confounding factors stated?	☐	☐	☐	+
7. Were the outcomes measured in a valid and reliable way?	+	☐	☐	☐
8. Was appropriate statistical analysis used?	+	☐	☐	☐

Overall appraisal: Include + Exclude ☐ Seek further info ☐

Comments (Including reason for exclusion)

© JBI, 2020. All rights reserved. JBI grants use of these tools for research purposes only. All other enquiries should be sent to jbisynthesis@adelaide.edu.au.

JBI CRITICAL APPRAISAL CHECKLIST FOR CASE CONTROL STUDIES

Reviewer: CSS and ESS Date: 24-06-2024

Author: Hosaka, T. et al Year: 1999 Record Number: 5

	Yes	No	Unclear	Not applicable
1. Were the groups comparable other than the presence of disease in cases or the absence of disease in controls?	+	☐	☐	☐
2. Were cases and controls matched appropriately?	☐	☐	+	☐
3. Were the same criteria used for identification of cases and controls?	+	☐	☐	☐
4. Was exposure measured in a standard, valid and reliable way?	+	☐	☐	☐
5. Was exposure measured in the same way for cases and controls?	+	☐	☐	☐
6. Were confounding factors identified?	☐	+	☐	☐
7. Were strategies to deal with confounding factors stated?	☐	☐	☐	+
8. Were outcomes assessed in a standard, valid and reliable way for cases and controls?	+	☐	☐	☐
9. Was the exposure period of interest long enough to be meaningful?	☐	☐	☐	+
10. Was appropriate statistical analysis used?	+	☐	☐	☐

Overall appraisal: Include + Exclude ☐ Seek further info ☐

Comments (Including reason for exclusion)

© JBI, 2020. All rights reserved. JBI grants use of these tools for research purposes only. All other enquiries should be sent to jbisynthesis@adelaide.edu.au.

JBI CRITICAL APPRAISAL CHECKLIST FOR ANALYTICAL CROSS SECTIONAL STUDIES

Reviewer: CSS and ESS Date: 24-06-2024

Author: Kim, M. K. and Alvi, A Year: 1999 Record Number: 6

	Yes	No	Unclear	Not applicable
1. Were the criteria for inclusion in the sample clearly defined?	☐	☐	+	☐
2. Were the study subjects and the setting described in detail?	+	☐	☐	☐
3. Was the exposure measured in a valid and reliable way?	+	☐	☐	☐
4. Were objective, standard criteria used for measurement of the condition?	☐	+	☐	☐
5. Were confounding factors identified?	☐	+	☐	☐
6. Were strategies to deal with confounding factors stated?	☐	☐	☐	+
7. Were the outcomes measured in a valid and reliable way?	☐	+	☐	☐
8. Was appropriate statistical analysis used?	☐	+	☐	☐

Overall appraisal: Include + Exclude ☐ Seek further info ☐

Comments (Including reason for exclusion)

© JBI, 2020. All rights reserved. JBI grants use of these tools for research purposes only. All other enquiries should be sent to jbisynthesis@adelaide.edu.au.

JBI CRITICAL APPRAISAL CHECKLIST FOR ANALYTICAL CROSS SECTIONAL STUDIES

Reviewer: CSS and ESS Date: 24-06-2024

Author: Lobb, E. A. et al Year: 2010 Record Number: 7

	Yes	No	Unclear	Not applicable
1. Were the criteria for inclusion in the sample clearly defined?	+	☐	☐	☐
2. Were the study subjects and the setting described in detail?	+	☐	☐	☐
3. Was the exposure measured in a valid and reliable way?	+	☐	☐	☐
4. Were objective, standard criteria used for measurement of the condition?	☐	+	☐	☐
5. Were confounding factors identified?	☐	+	☐	☐
6. Were strategies to deal with confounding factors stated?	☐	☐	☐	+
7. Were the outcomes measured in a valid and reliable way?	☐	☐	+	☐
8. Was appropriate statistical analysis used?	☐	+	☐	☐

Overall appraisal: Include + Exclude ☐ Seek further info ☐

Comments (Including reason for exclusion)

© JBI, 2020. All rights reserved. JBI grants use of these tools for research purposes only. All other enquiries should be sent to jbisynthesis@adelaide.edu.au.

JBI CRITICAL APPRAISAL CHECKLIST FOR ANALYTICAL CROSS SECTIONAL STUDIES

Reviewer: CSS and ESS Date: 24-06-2024

Author: Magro, E. et al Year: 2016 Record Number: 8

	Yes	No	Unclear	Not applicable
1. Were the criteria for inclusion in the sample clearly defined?	+	☐	☐	☐
2. Were the study subjects and the setting described in detail?	+	☐	☐	☐
3. Was the exposure measured in a valid and reliable way?	+	☐	☐	☐
4. Were objective, standard criteria used for measurement of the condition?	☐	+	☐	☐
5. Were confounding factors identified?	☐	+	☐	☐
6. Were strategies to deal with confounding factors stated?	☐	☐	☐	+
7. Were the outcomes measured in a valid and reliable way?	+	☐	☐	☐
8. Was appropriate statistical analysis used?	☐	+	☐	☐

Overall appraisal: Include + Exclude ☐ Seek further info ☐

Comments (Including reason for exclusion)

© JBI, 2020. All rights reserved. JBI grants use of these tools for research purposes only. All other enquiries should be sent to jbisynthesis@adelaide.edu.au.

JBI CRITICAL APPRAISAL CHECKLIST FOR ANALYTICAL CROSS SECTIONAL STUDIES

Reviewer: CSS and ESS Date: 24-06-2024

Author: Malmstrom, A. et al Year: 2020 Record Number: 9

	Yes	No	Unclear	Not applicable
1. Were the criteria for inclusion in the sample clearly defined?	+	☐	☐	☐
2. Were the study subjects and the setting described in detail?	+	☐	☐	☐
3. Was the exposure measured in a valid and reliable way?	+	☐	☐	☐
4. Were objective, standard criteria used for measurement of the condition?	☐	+	☐	☐
5. Were confounding factors identified?	+	☐	☐	☐
6. Were strategies to deal with confounding factors stated?	+	☐	☐	☐
7. Were the outcomes measured in a valid and reliable way?	☐	+	☐	☐
8. Was appropriate statistical analysis used?	☐	+	☐	☐

Overall appraisal: Include + Exclude ☐ Seek further info ☐

Comments (Including reason for exclusion)

© JBI, 2020. All rights reserved. JBI grants use of these tools for research purposes only. All other enquiries should be sent to jbisynthesis@adelaide.edu.au.

JBI CRITICAL APPRAISAL CHECKLIST FOR ANALYTICAL CROSS SECTIONAL STUDIES

Reviewer: CSS and ESS Date: 24-06-2024

Author: Motlagh, A. et al Year: 2014 Record Number: 10

	Yes	No	Unclear	Not applicable
1. Were the criteria for inclusion in the sample clearly defined?	+	☐	☐	☐
2. Were the study subjects and the setting described in detail?	☐	+	☐	☐
3. Was the exposure measured in a valid and reliable way?	+	☐	☐	☐
4. Were objective, standard criteria used for measurement of the condition?	☐	+	☐	☐
5. Were confounding factors identified?	☐	☐	+	☐
6. Were strategies to deal with confounding factors stated?	☐	☐	☐	+
7. Were the outcomes measured in a valid and reliable way?	+	☐	☐	☐
8. Was appropriate statistical analysis used?	+	☐	☐	☐

Overall appraisal: Include + Exclude ☐ Seek further info ☐

Comments (Including reason for exclusion)

© JBI, 2020. All rights reserved. JBI grants use of these tools for research purposes only. All other enquiries should be sent to jbisynthesis@adelaide.edu.au.

JBI CRITICAL APPRAISAL CHECKLIST FOR ANALYTICAL CROSS SECTIONAL STUDIES

Reviewer: CSS and ESS Date: 24-06-2024

Author: Nwankwo, K. C. et al Year: 2013 Record Number: 11

	Yes	No	Unclear	Not applicable
1. Were the criteria for inclusion in the sample clearly defined?	☐	+	☐	☐
2. Were the study subjects and the setting described in detail?	+	☐	☐	☐
3. Was the exposure measured in a valid and reliable way?	+	☐	☐	☐
4. Were objective, standard criteria used for measurement of the condition?	☐	+	☐	☐
5. Were confounding factors identified?	☐	+	☐	☐
6. Were strategies to deal with confounding factors stated?	☐	☐	☐	+
7. Were the outcomes measured in a valid and reliable way?	+	☐	☐	☐
8. Was appropriate statistical analysis used?	+	☐	☐	☐

Overall appraisal: Include + Exclude ☐ Seek further info ☐

Comments (Including reason for exclusion)

© JBI, 2020. All rights reserved. JBI grants use of these tools for research purposes only. All other enquiries should be sent to jbisynthesis@adelaide.edu.au.

JBI CRITICAL APPRAISAL CHECKLIST FOR ANALYTICAL CROSS SECTIONAL STUDIES

Reviewer: CSS and ESS Date: 24-06-2024

Author: Perera M.C. et al Year: 2013 Record Number: 12

	Yes	No	Unclear	Not applicable
1. Were the criteria for inclusion in the sample clearly defined?	☐	+	☐	☐
2. Were the study subjects and the setting described in detail?	☐	+	☐	☐
3. Was the exposure measured in a valid and reliable way?	☐	☐	+	☐
4. Were objective, standard criteria used for measurement of the condition?	☐	+	☐	☐
5. Were confounding factors identified?	☐	+	☐	☐
6. Were strategies to deal with confounding factors stated?	☐	☐	☐	+
7. Were the outcomes measured in a valid and reliable way?	☐	+	☐	☐
8. Was appropriate statistical analysis used?	☐	+	☐	☐

Overall appraisal: Include + Exclude ☐ Seek further info ☐

Comments (Including reason for exclusion)

© JBI, 2020. All rights reserved. JBI grants use of these tools for research purposes only. All other enquiries should be sent to jbisynthesis@adelaide.edu.au.

JBI CRITICAL APPRAISAL CHECKLIST FOR ANALYTICAL CROSS SECTIONAL STUDIES

Reviewer: CSS and ESS Date: 24-06-2024

Author: Salander, P. and Spetz Year: 2002 Record Number: 13

	Yes	No	Unclear	Not applicable
1. Were the criteria for inclusion in the sample clearly defined?	+	☐	☐	☐
2. Were the study subjects and the setting described in detail?	+	☐	☐	☐
3. Was the exposure measured in a valid and reliable way?	☐	☐	+	☐
4. Were objective, standard criteria used for measurement of the condition?	☐	+	☐	☐
5. Were confounding factors identified?	☐	+	☐	☐
6. Were strategies to deal with confounding factors stated?	☐	☐	☐	+
7. Were the outcomes measured in a valid and reliable way?	☐	+	☐	☐
8. Was appropriate statistical analysis used?	☐	+	☐	☐

Overall appraisal: Include + Exclude ☐ Seek further info ☐

Comments (Including reason for exclusion)

© JBI, 2020. All rights reserved. JBI grants use of these tools for research purposes only. All other enquiries should be sent to jbisynthesis@adelaide.edu.au.

JBI CRITICAL APPRAISAL CHECKLIST FOR ANALYTICAL CROSS SECTIONAL STUDIES

Reviewer: CSS and ESS Date: 24-06-2024

Author: Umeda, M. et al Year: 2003 Record Number: 14

	Yes	No	Unclear	Not applicable
1. Were the criteria for inclusion in the sample clearly defined?	☐	+	☐	☐
2. Were the study subjects and the setting described in detail?	☐	+	☐	☐
3. Was the exposure measured in a valid and reliable way?	☐	☐	+	☐
4. Were objective, standard criteria used for measurement of the condition?	☐	+	☐	☐
5. Were confounding factors identified?	☐	+	☐	☐
6. Were strategies to deal with confounding factors stated?	☐	☐	☐	+
7. Were the outcomes measured in a valid and reliable way?	+	☐	☐	☐
8. Was appropriate statistical analysis used?	☐	+	☐	☐

Overall appraisal: Include + Exclude ☐ Seek further info ☐

Comments (Including reason for exclusion)

© JBI, 2020. All rights reserved. JBI grants use of these tools for research purposes only. All other enquiries should be sent to jbisynthesis@adelaide.edu.au.

JBI CRITICAL APPRAISAL CHECKLIST FOR ANALYTICAL CROSS SECTIONAL STUDIES

Reviewer: CSS and ESS Date: 24-06-2024

Author: Wang, D.C. et al Year: 2011 Record Number: 15

	Yes	No	Unclear	Not applicable
1. Were the criteria for inclusion in the sample clearly defined?	+	☐	☐	☐
2. Were the study subjects and the setting described in detail?	+	☐	☐	☐
3. Was the exposure measured in a valid and reliable way?	+	☐	☐	☐
4. Were objective, standard criteria used for measurement of the condition?	☐	+	☐	☐
5. Were confounding factors identified?	☐	☐	+	☐
6. Were strategies to deal with confounding factors stated?	☐	☐	☐	+
7. Were the outcomes measured in a valid and reliable way?	+	☐	☐	☐
8. Was appropriate statistical analysis used?	+	☐	☐	☐

Overall appraisal: Include + Exclude ☐ Seek further info ☐

Comments (Including reason for exclusion)

© JBI, 2020. All rights reserved. JBI grants use of these tools for research purposes only. All other enquiries should be sent to jbisynthesis@adelaide.edu.au.

JBI CRITICAL APPRAISAL CHECKLIST FOR ANALYTICAL CROSS SECTIONAL STUDIES

Reviewer: CSS and ESS Date: 24-06-2024

Author: Yamamoto, F. et al Year: 2011 Record Number: 16

	Yes	No	Unclear	Not applicable
1. Were the criteria for inclusion in the sample clearly defined?	+	☐	☐	☐
2. Were the study subjects and the setting described in detail?	+	☐	☐	☐
3. Was the exposure measured in a valid and reliable way?	+	☐	☐	☐
4. Were objective, standard criteria used for measurement of the condition?	☐	+	☐	☐
5. Were confounding factors identified?	☐	☐	+	☐
6. Were strategies to deal with confounding factors stated?	☐	☐	☐	+
7. Were the outcomes measured in a valid and reliable way?	+	☐	☐	☐
8. Was appropriate statistical analysis used?	+	☐	☐	☐

Overall appraisal: Include + Exclude ☐ Seek further info ☐

Comments (Including reason for exclusion)

© JBI, 2020. All rights reserved. JBI grants use of these tools for research purposes only. All other enquiries should be sent to jbisynthesis@adelaide.edu.au.
